# A unified approach for allele frequency estimation, SNP detection and association studies based on pooled sequencing data using EM algorithms

**DOI:** 10.1186/1471-2164-14-S1-S1

**Published:** 2013-01-21

**Authors:** Quan Chen, Fengzhu Sun

**Affiliations:** 1 Molecular and Computational Biology Program, University of Southern California, Los Angeles, CA 90089-2910, USA; 2 TNLIST/Department of Automation, Tsinghua University, Beijing 100084, PR China

## Abstract

**Background:**

Genome-wide association studies (GWAS) have identified many common polymorphisms associated with complex traits. However, these associated common variants explain only a small fraction of the phenotypic variances, leaving a substantial portion of genetic heritability unexplained. As a result, searches for "missing" heritability are drawing increasing attention, particularly for rare variant studies that often require a large sample size and, thus, extensive sequencing effort. Although the development of next generation sequencing (NGS) technologies has made it possible to sequence a large number of reads economically and efficiently, it is still often cost prohibitive to sequence thousands of individuals that are generally required for association studies. A more efficient and cost-effective design would involve pooling the genetic materials of multiple individuals together and then sequencing the pools, instead of the individuals. This pooled sequencing approach has improved the plausibility of association studies for rare variants, while, at the same time, posed a great challenge to the pooled sequencing data analysis, essentially because individual sample identity is lost, and NGS sequencing errors could be hard to distinguish from low frequency alleles.

**Results:**

A unified approach for estimating minor allele frequency, SNP calling and association studies based on pooled sequencing data using an expectation maximization (EM) algorithm is developed in this paper. This approach makes it possible to study the effects of minor allele frequency, sequencing error rate, number of pools, number of individuals in each pool, and the sequencing depth on the estimation accuracy of minor allele frequencies. We show that the naive method of estimating minor allele frequencies by taking the fraction of observed minor alleles can be significantly biased, especially for rare variants. In contrast, our EM approach can give an unbiased estimate of the minor allele frequency under all scenarios studied in this paper. A SNP calling approach, EM-SNP, for pooled sequencing data based on the EM algorithm is then developed and compared with another recent SNP calling method, SNVer. We show that EM-SNP outperforms SNVer in terms of the fraction of db-SNPs among the called SNPs, as well as transition/transversion (Ti/Tv) ratio. Finally, the EM approach is used to study the association between variants and type I diabetes.

**Conclusions:**

The EM-based approach for the analysis of pooled sequencing data can accurately estimate minor allele frequencies, call SNPs, and find associations between variants and complex traits. This approach is especially useful for studies involving rare variants.

## Introduction

Finding genomic variants associated with complex traits is one of the most important problems in modern genomics. Genome-wide association studies (GWAS) based on common variants have been the dominant approach to achieve this objective [1]. However, the genomic variants identified in GWAS studies often explain only a small portion of the phenotypic variation related to heritable human diseases, a phenomenon known as "missing heritability" in genomics literature [2]. This missing heritability problem has led to increasingly skeptical views of the common disease-common variant (CD-CV) hypothesis which predicts that common disease-causing alleles, or variants, will be found in all human populations that manifest a given disease. On the other hand, interest in studies on rare variants with minor allele frequencies less than 1% is growing [3,4].

Studies of rare variants are complicated by the low minor allele frequencies of rare variants. The development of next generation sequencing (NGS) technologies such as Illumina and Roche 454 has made it possible to sequence a large number of reads economically. Despite such important progress, sequencing a large number of individuals separately is still costly for most biological laboratories. One frequently adopted approach to reduce sequencing cost in the search of rare variants is pooled sequencing, where mixtures of genetic materials from several individuals are grouped together to form a pool for a single sequencing. While this design greatly lowers the sequencing cost, it also makes it hard to distinguish true genetic polymorphisms from sequencing errors, estimate minor allele frequencies at the polymorphic loci, and perform association studies on the rare variants.

Several research groups have used pooled sequencing to detect rare variants that are associated with complex traits such as retinitis pigmentosa, diabetes, cancer, and inflammatory bowel disease [5-8]. There are generally two types of pooling designs. One is pooling of tagged samples with each individual tagged by a unique short barcode. In this design, the genomic origins of the reads can be identified. However, barcoding many individuals and distinguishing these barcodes from each other can still be a challenging task. The second type of pooling is to mix the genetic materials from different individuals without tagging, and then generate reads from the mixture of genetic materials using NGS. With this design, the identities of the individuals from whom the reads originate cannot be identified. In this paper, we concentrate on the second type of pooling design.

Several groups have developed SNP calling methods based on pooled sequencing data [7,9-12]. Out *et al.*[7] modeled the number of sequencing errors as a Poisson random variable and identified SNPs by comparing the number of minor alleles within the reads with the Poisson distribution. For rare variants with minor allele frequencies similar to or lower than the sequencing error rate, this approach could miss many true variants if the pool size is relatively large. Druley *et al.*[9] developed a SNP identification method, SNPSeeker, that can be applied to large pools by using control sequences without SNPs. In many studies, control sequences may not be available, making this approach impractical. Also, the program can only be used to analyze Illumina data. Bansal *et al.*[10] developed a method called CRISP to identify rare variants by comparing the minor allele frequencies across multiple pools using contingency tables. It was shown that CRISP outperforms SNPSeeker in terms of accuracy, but CRISP is more computationally demanding. Altmann *et al.*[13] improved the computational speed of CRISP and identified SNPs as the variants with different minor allele frequencies across at least two pools. Wei *et al.*[11] developed a statistical tool, called SNVer, for variant identification. For each pool, SNVer first defined a p-value by testing the hypothesis that the minor allele frequency is above a given threshold and then combined the p-values for individual pools to give an overall p-value using Simes methods as in [12]. This algorithm makes it possible to rank the observed variants so that the top ranked ones are more likely to be true SNPs. However, the algorithm needs a pre-specified sequencing error rate which can be difficult to do because the sequencing error rates can be position dependent. In the above studies, the investigators are mainly concerned with the detection of SNPs; they do not aim to estimate minor allele frequencies.

In order to estimate minor allele frequencies in pooling studies, several groups developed statistical models for the sampling of individuals and the sampling of reads from the individuals in the pools [14,15]. These studies assumed a pre-defined constant sequencing error rate across different loci. However, sequencing error rates can vary for different loci depending on the nucleotide contents of the surrounding genomic regions. The effects of mis-specifying the sequencing error rate on minor allele frequency estimation, SNP detection and power of association studies are not clear. Using similar models, Lee *et al*. [16] studied an optimal design in pooling studies. This involved the number of individuals in each pool and the number of pools. Recently, Chen *et al.*[17] considered more complex issues such as uneven sampling of individuals, different coverage of the minor and major alleles due to either PCR amplification or reads mapping, and reads quality scores.

In this paper, we develop new methods for estimating minor allele frequencies, SNP detection, and association studies using pooled sequencing data based on the models in [14-16]. In contrast to methods developed in previous studies, we do not assume that the sequencing error rate is constant. Instead, we estimate the sequencing error rate for each position together with the minor allele frequency based on the minor and major allele counts for all the pools using an expectation-maximization (EM) algorithm. We show that the naive estimation of the allele frequency by the fraction of minor alleles in the reads can be significantly inflated, especially for rare variants, while the EM approach can give an unbiased estimate of the minor allele frequency in all situations we studied. The estimation accuracy of the EM algorithm increases with the number of reads and the number of pools, but decreases with the number of chromosomes in each pool. Based on the allele frequency estimation, we develop a SNP calling method, EM-SNP, and an association test using likelihood ratio statistics. The likelihood ratio statistic used in EM-SNP is then used to rank candidate polymorphic loci to determine if they are true polymorphisms. Using a real re-sequencing dataset, we show that, for rare variants with minor allele frequencies lower than 1%, the fraction of dbSNPs (http://www.ncbi.nlm.nih.gov/projects/SNP/) among the SNPs called by EM-SNP is higher than that of SNVer. Similarly, the transition/transversion ratio of rare variants called by EM-SNP is higher than that of SNVer. These observations show that EM-SNP outperforms SNVer at calling rare variants with minor allele frequencies less than 1%.

## Materials and methods

### Notation

Consider a locus along the genome. Let *f *be the frequency of the minor allele (denoted as "1"), and 1 - *f *be the frequency of the major allele (denoted as "0") in a population. We also consider the following potential sequencing error rates:

P(1|0)=α,P(0|1)=β.

Assume that a total of *G *pools of individuals are sequenced and each pool contains *K/*2 individuals (*K *chromosomes). For each pool *g*, a total of *n_g _*reads are mapped to this locus, with $n_{0_{g}}$ reads carrying the major allele and $n_{1_{g}}$ reads carrying the minor allele. Thus $n_{g}={n_{0}}_{{}_{g}}+n_{1_{g}}$.

Let *C *be the number of chromosomes carrying the minor allele among the *K *chromosomes in a pool. Then *C *follows a binomial distribution, i.e

$$\begin{array}{c} P\left\{C=k\right\}=\text{Bin}\left(k;K,f\right)=\left(\begin{array}{c} K\\ k \end{array}\right)f^{k}{\left(1-f\right)}^{K-k},\\ \;\;\;\;\;\;\;\;\;k=0,1,2,\cdot \cdot \cdot ,K. \end{array}$$

Conditional on *C *= *k*, the probability that a sequence read covering a variant carries the minor allele is

$$p_{k}=\frac{k}{K}\left(1-\beta \right)+\frac{K-k}{K}\alpha .$$

Thus, the probability of observing the data for the *g*-th pool is,

(1) $$P_{g}\left(n_{0_{g}},n_{1_{g}}|f,\alpha ,\beta \right)=\overset{K}{\underset{k=0}{\sum }}\text{Bin}\left(n_{1_{g}};n_{g},p_{k}\right)\text{Bin}\left(k;K,f\right).$$

Since the pools can be considered independent, the likelihood of observing the data for all the pools is

$$L\left(f,\alpha ,\beta \right)=\overset{G}{\underset{g=1}{\prod }}P_{g}\left(n_{0_{g}},n_{1_{g}}|f,\alpha ,\beta \right).$$

Given the above likelihood expression and the data $\left\{\left(n_{0_{g}},n_{1_{g}}\right),g=1,2,\cdot \cdot \cdot ,G\right\}$, our objectives are as follows

• Find the maximum likelihood estimate of (*f, α, β*).

• Determine whether an observed variant is a true SNP or not, i.e. SNP calling.

• Find the variants associated with a phenotype of interest.

### Computational methods

#### An expectation-maximization (EM) approach for allele frequency estimation

Based on the likelihood function, an approximate solution to the maximum likelihood estimation of the parameters can be obtained using the EM algorithm. We consider the following missing data:

• *C_g_*: the number of chromosomes carrying the minor allele in the *g*-th pool;

• *I_gi_*: the true underlying allele state (major (0) or minor (1)) of read *i *in the *g*-th pool;

• *r_gi_*: the observed allele state (major (0) and minor (1)) of the *i*-th read in the *g*-th pool.

We also use the following notation:

$$\begin{array}{c} C=\sum_{g=1}^{G}C_{g},\\ T_{g}=\sum_{i=1}^{n_{g}}I_{gi},\\ T_{11}=\sum_{g=1}^{G}\sum_{i=1}^{n_{g}}I_{gi}r_{gi},\\ T_{10}=\sum_{g=1}^{G}\sum_{i=1}^{n_{g}}I_{gi}\left(1-r_{gi}\right),\\ T_{01}=\sum_{g=1}^{G}\sum_{i=1}^{n_{g}}\left(1-I_{gi}\right)r_{gi},\\ T_{00}=\sum_{g=1}^{G}\sum_{i=1}^{n_{g}}\left(1-I_{gi}\right)\left(1-r_{gi}\right). \end{array}$$

Based on the above notation, the complete log-likelihood is:

(2) $$\begin{array}{c} \;\;\;\;\log P\left(T_{ij}^{\left(g\right)},C_{g},n_{1_{g}},n_{0_{g}},g=1,\cdot \cdot \cdot ,G|f,\alpha ,\beta \right)\\ =C\log f+\left(GK-C\right)\log\left(1-f\right)\\ \;+\overset{G}{\underset{g=1}{\sum }}\left\{\log\left(\begin{array}{c} k\\ C_{g} \end{array}\right)\left(\begin{array}{c} n_{g}\\ n_{1_{g}} \end{array}\right)\left(\begin{array}{c} n_{1_{g}}\\ T_{01}^{\left(g\right)} \end{array}\right)\left(\begin{array}{c} n_{0_{g}}\\ T_{10}^{\left(g\right)} \end{array}\right)\right)+T_{1.}^{\left(g\right)}\log\frac{C_{g}}{K}\\ \;+\left(n_{g}-T_{1.}^{\left(g\right)}\right)\log\left(\left(1-\frac{C_{g}}{K}\right)\right\}+T_{11}\log\left(1-\beta \right)\\ \;+T_{10}\log\beta +T_{01}\log\alpha +T_{00}\log\left(1-\alpha \right). \end{array}$$

Suppose that the value of Θ = (*f, α, β*) at the *t*-th iteration is Θ^(*t*) ^= (*f*^(*t*)^, *α*^(*t*)^, *β*^(*t*)^). The maximization (M)-step gives:

$$\begin{array}{c} f^{\left(t+1\right)}=\frac{E_{\left(t\right)}\left(C\right)}{G\times K},\\ \alpha^{\left(t+1\right)}=\frac{E_{\left(t\right)}\left(T_{01}\right)}{E_{\left(t\right)}\left(T_{01}\right)+E_{\left(t\right)}\left(T_{00}\right)},\\ \beta^{\left(t+1\right)}=\frac{E_{\left(t\right)}\left(T_{10}\right)}{E_{\left(t\right)}\left(T_{10}\right)+E_{\left(t\right)}\left(T_{11}\right)}. \end{array}$$

Note the expectation *E*_(*t*) _is taken when the parameters are at Θ^(*t*)^.

The expectation (E)-step is formulated as follows:

(3) $$\begin{array}{c} \;E_{\left(t\right)}\left(C_{g}|\text{Data}\right)\\ =\frac{\sum_{k=0}^{K}k\left(\begin{array}{c} K\\ k \end{array}\right)f^{k}{\left(1-f\right)}^{K-k}\left(\begin{array}{c} n_{g}\\ n_{1_{g}} \end{array}\right){\left(p_{k}\right)}^{n_{1_{g}}}{\left(1-p_{k}\right)}^{n_{0_{g}}}}{\sum_{k=0}^{K}\left(\begin{array}{c} K\\ k \end{array}\right)f^{k}{\left(1-f\right)}^{K-k}\left(\begin{array}{c} n_{g}\\ n_{1_{g}} \end{array}\right){\left(p_{k}\right)}^{n_{1_{g}}}{\left(1-p_{k}\right)}^{n_{0_{g}}}}, \end{array}$$

and

(4) $$E_{\left(t\right)}\left(C|\text{Data}\right)=\overset{G}{\underset{g=1}{\sum }}E_{\left(t\right)}\left(C_{g}|\text{Data}\right),$$

where all the parameters in the equations are of the values taken at the *t*-th step.

From Equations 3 and 4, we are able to obtain the recursive formula for *f*.

Next we calculate *E*_(*t*)_(*T*_11_|Data). Note that

$$\begin{array}{c} \;E_{\left(t\right)}\left(I_{gi}r_{gi}|\text{Data}\right)\\ =\frac{\sum_{k=0}^{K}\frac{k}{K}\left(1-\beta \right)\text{Bin}\left(n_{1_{g}}-1;n_{g}-1,p_{k}\right)\text{Bin}\left(k;K,f\right)}{P\left(\left(n_{0_{g}},n_{1_{g}}\right)\right)}, \end{array}$$

which does not depend on *i*, and we denote it as $E\left(I_{g}r_{g}|\left(n_{0_{g}},n_{{\text{1}}_{g}}\right)\right)$. The denominator $P\left(\left(n_{0_{g}},n_{{\text{1}}_{g}}\right)\right)$ can be calculated from Equation 1. Thus,

(5) $$E_{\left(t\right)}\left(T_{11}|\text{Data}\right)=\overset{G}{\underset{g=1}{\sum }}n_{g}E\left(I_{g}r_{g}|\left(n_{0_{g}},n_{1_{g}}\right)\right).$$

Similarly, we can derive the formulas for *E*_(*t*)_(*T*_10_|Data), *E*_(*t*)_(*T*_01_|Data) and *E*_(*t*)_(*T*_00_|Data), and the recursive formulas for *α *and *β *can be derived from them.

#### SNP identification using EM

Due to sequencing errors, the observed variants may contain a significant amount of false positives, *i.e*. loci that are not truly polymorphic. Thus, before testing for associations with phenotypes, we need to determine the true polymorphic sites. This step is especially important in the case of rare variants since the sequencing error rates for NGS could be close to or even higher than the minor allele frequencies.

Consider a case-control study with a group of case individuals and another group of control individuals. Let *f*_1 _and *f*_0 _be the minor allele frequencies at a locus among the cases and controls, respectively. Denote **f **= (*f*_0_, *f*_1_) and **0 **= (0, 0). We can test if an observed variant is a true SNP using the likelihood ratio test for *H*_0 _: *f*_0 _= *f*_1 _= 0 vs. *H*_1 _: *f*_0 _≠ 0 or *f*_1 _≠ 0:

(6) $$\Lambda =2\left(l_{f\neq 0}-l_{f=0}\right)\overset{H_{0}}{\sim }\frac{1}{4}I_{0}+\frac{1}{2}\chi_{1}^{2}+\frac{1}{4}\chi_{2}^{2},$$

where *l*_f _is the maximum log-likelihood of the observed data for both the cases and the controls. Note that the null hypothesis **f **= **0 **is on the boundary of the region of the parameters of interest. Therefore, the asymptotic distribution of Λ is $\frac{1}{4}I_{0}+\frac{1}{2}\chi_{1}^{2}+\frac{1}{4}\chi_{2}^{2}$ when the number of pools is large according to [18], where *I*_0 _is the point mass at 0 and $\chi_{i}^{2}$, *i *= 1, 2 are the chi-square distributions with *i *degrees of freedom. When the number of pools is relatively small, simulation approaches for the null distribution of Λ are needed to obtain the asymptotic distribution.

We can also test if an observed variant is a true SNP using cases or controls separately. For the control pools, we conduct a likelihood ratio test for *H*_0 _: *f*_0 _= 0 vs. *H*_1 _: *f*_0 _> 0. Similarly, we replace *f*_0 _by *f*_1 _for the case pools. We then use the statistic

(7) $$\Lambda_{i}=2\left(l_{f_{i}\neq 0}-l_{f_{i}=0}\right)\overset{H_{0}}{\sim }\frac{1}{2}I_{0}+\frac{1}{2}\chi_{1}^{2},\;\;i=1,2,$$

to test each hypothesis, where $l_{f_{\text{1}}}$ and $l_{f_{\text{0}}}$ are the maximum log-likelihood of the data for the cases and controls, respectively. Because the null hypothesis *f_i _*= 0 is on the boundary of parameter region *f_i _*> 0, the statistic Λ*_i _*has an asymptotic distribution $\frac{1}{2}I_{0}+\frac{1}{2}\chi_{1}^{2}$ when the number of pools is large according to [18]. We refer to the above method for SNP identification as EM-SNP.

#### Testing for associations between a SNP and a phenotype in case-control studies

We test if a SNP is associated with a phenotype of interest using the likelihood ratio test again. Here we test the alternative hypothesis *H*_1 _: *f*_1 _≠ *f*_0 _versus the null hypothesis *H*_0 _: *f*_1 _= *f*_0_. This association test is conducted by the likelihood ratio test statistic:

(8) $$\Lambda =2\left[l\left(\text{unrestricted}\;{\hat{f}}_{0},{\hat{f}}_{1},\hat{\alpha },\hat{\beta }\right)-l\left(\text{restricted}\;\hat{f},\hat{\alpha },\hat{\beta }\right)\right]\overset{H_{0}}{\sim }\chi_{1}^{2}.$$

This statistic has an asymptotic chi-square distribution with 1 degree of freedom.

### Simulation studies

We use simulations to evaluate our approaches for allele frequency estimation, SNP detection and test for association. A large range of parameter space is considered to see how different parameters affect the performance of our methods. These parameters include minor allele frequency (*f*), sequencing error rate (*α*), the number of chromosomes in each pool (*K*), the number of pools (*G*) and the relative risk for a disease (*λ*).

#### Pooled data generation

In our simulations, we set *α *= *β *and choose four starting values of *α *= *β *= 0.05%, 0.1%, 0.5%, 1% corresponding to different sequencing error rates ranging from low to high. The sequencing error rate for current NGS technologies is around 1% and we expect that it will decrease as the technologies improve. Therefore, we also consider much lower sequencing error rate in our studies. For the allele frequency, we choose four values *f *= 0.1%, 0.5%, 1%, 5%. Loci with minor allele frequencies above 5% are considered to be common. We want to study if it is possible to estimate the minor allele frequency even if it is lower than the sequencing error rate. We let the read number *n *= 1000 and 3000, which is around the sequencing depth in [8]. To study the effect of the number of chromosomes, we let *K *= 50, 100, 200. The number of pools is set at *G *= 10, 20, 50.

Since the sequencing error rate can vary from locus to locus and from one pool to another, we generate 1000 *α_i_*(= *β_i_*, *i *= 1, · · ·, 1000) values from a normal distribution with mean equal to the starting values of *α*, and variance equal to 0.1 times the starting values. Finally, we generate 1000 pooled data sets with each combination of the five parameters (*K*, *G*, *n*, *f*, *α*) based on *α_i_*(= *β_i_*), *i *= 1, ⋯, 1000.

#### Measuring the accuracy of the allele frequency estimation

For each of the 4 × 4 × 2 × 3 × 3 = 288 combinations, we do the following:

1. In the *i*-th simulation, we use the EM algorithm derived above to estimate the parameters (*f*, *α*, *β*), denoted as $\left({\hat{f}}_{\text{em}}^{\left(i\right)},\hat{\alpha_{i}},\hat{\beta_{i}}\right)$. We also consider a naive estimate for the minor allele frequency as the fraction of observed minor alleles in the observed reads,

(9) $${\hat{f}}_{\text{avg}}^{\left(i\right)}=\frac{\sum_{g=1}^{G}n_{1_{g}}}{\sum_{g=1}^{G}n_{g}}.$$

2. Repeat Step 1) for R = 1000 times.

3. Compute the mean squared error (MSE) of ${\hat{f}}_{\text{em}}$ and ${\hat{f}}_{\text{avg}}$ from the true population minor allele frequency *f*,

$$\begin{array}{c} MSE\left({\hat{f}}_{\text{em}}\right)=\frac{1}{R}\overset{R}{\underset{i=1}{\sum }}{\left({\hat{f}}_{\text{em}}^{\left(i\right)}-f\right)}^{2},\\ MSE\left({\hat{f}}_{\text{avg}}\right)=\frac{1}{R}\overset{R}{\underset{i=1}{\sum }}{\left({\hat{f}}_{\text{avg}}^{\left(i\right)}-f\right)}^{2}. \end{array}$$

4. Compute the MSE of ${\hat{f}}_{\text{em}}$ and ${\hat{f}}_{\text{avg}}$ from the fraction of chromosomes carrying minor alleles *f*_frac _= *C*/(*KG*) in the pools,

$$\begin{array}{c} Cg\left({\hat{f}}_{\text{em}}\right)=\frac{1}{R}\overset{R}{\underset{i=1}{\sum }}{\left({\hat{f}}_{\text{em}}^{\left(i\right)}-f_{\text{frac}}\right)}^{2},\\ Cg\left({\hat{f}}_{\text{avg}}\right)=\frac{1}{R}\overset{R}{\underset{i=1}{\sum }}{\left({\hat{f}}_{\text{avg}}^{\left(i\right)}-f_{\text{frac}}\right)}^{2}. \end{array}$$

We use both MSE and Cg to compare the accuracy of the EM algorithm with the naive approach of estimating *f *by the fraction of reads carrying the minor allele.

#### Generating case-control data to study the power of SNP identification and association studies using EM

In order to evaluate the power of SNP identification using EM-SNP and test for association, we simulate case-control data as follows. When generating the control data, we assume that the minor allele frequency is *f *and that the locus is under Hardy-Weinberg equilibrium. When generating the genotypes of the case individuals, we assume that the penetrance (the probability an individual is affected) of the genotypes 00, 01 and 11 are *g*_0 _= 0.01, *λg*_0_, and λ^2^*g*_0_, respectively. In our simulations, we choose *λ *= 1.2, 2, and 4.

We can use the case or control samples separately or combine them for SNP detection as in the "SNP identification using EM" subsection. For example, we consider both the cases and controls jointly. The log-likelihood ratio statistic Λ (or Λ*_i _*if we use case or control samples separately) defined in equation 6 is used to test if an observed variant is a true polymorphic locus or not. For a given type I error *γ *(= 0.05 in our study), we claim that the variant is truly polymorphic if Λ >*t_γ_*, where *t_γ _*is the threshold corresponding to type I error *γ*. If the threshold can not be found theoretically, we can do parametric bootstrap to find the threshold. Firstly, assume that the variant site is not polymorphic, estimate the allele frequency and error rate using the maximum likelihood approach. Secondly, generate the reads data as in our model a large number of times (*R *= 1000), and obtain the empirical distribution of Λ. For a given type I error *γ*, we find the upper *γ *percentile *t_γ_*. Finally, the null hypothesis is rejected if the value of Λ is at least *t_γ_*. For a given relative risk *λ*, we repeat these steps 1000 times and the power is the fraction of times that the locus is called as polymorphic.

Similar approaches can be used to study the power of association studies using the pooling design. For details, see additional file 1.

### A pooled sequencing data set related to type 1 diabetes [8]

We use our method to study the pooled sequencing data related to type 1 Diabetes dataset (T1D) in [8] and compare the results with current methods for SNP identifiction [11]. The data was generated using DNA samples of 480 T1D patients and 480 healthy controls, arranged in 20 DNA pools, with 48 patients/controls in each pool. Roche 454 sequencing was used to sequence 144 target regions that cover exons and splice sites of 10 candidate genes. We use MOSAIK (http://bioinformatics.bc.edu/marthlab/Mosaik) to map the reads to the human reference genome (hg19) with parameters -hs 15 -p 12 -mmp 0.05 -act 26 - mhp 100 -bw 51 as recommended in its documentation. MOSAIK is a widely used reference-guided assembler that hashes the whole reference genome and locate information in the hash table using a 'jump database' [19-21]. Then we use SAMtools (http://samtools.sourceforge.net/) [22] to pileup the reads onto the target regions. We also remove indels and keep loci that are covered by at least one read in each pool. Finally, we use ANNOVAR [23] to annotate the identified SNPs.

## Results

We first present our results on the effects of various parameters on the estimation accuracy of the minor allele frequency using the EM algorithm. We then present the results on the power of SNP detection and association studies. Finally, we present our results on the analysis of the data in [8] using the approaches in the "Materials and Methods" section.

### The effects of minor allele frequency, sequencing error rate, number of individuals in the pools and number of pools on the accuracy of allele frequency estimation

We compare our EM estimate ${\hat{f}}_{\text{em}}$ with the naive estimate ${\hat{f}}_{\text{avg}}$ for minor allele frequency *f*. Table 1 gives a brief summary of the comparisons between these two methods. Each cell corresponds to the number of scenarios that the mean squared error (using either MSE or Cg) of ${\hat{f}}_{\text{em}}$ exceeds ${\hat{f}}_{\text{avg}}$. It shows that ${\hat{f}}_{\text{em}}$ consistently outperforms ${\hat{f}}_{\text{avg}}$ whenever *f *≤ 0.1% or (*f *≤ 1%, *α *≥ 0.5%), which covers the typical situations of rare variant studies under current NGS technologies. Moreover, the advantage of the EM method increases as allele frequency *f *decreases and as sequencing error rate *α *increases, which is reasonable since it becomes more difficult for a naive estimate such as ${\hat{f}}_{\text{avg}}$ to distinguish true minor alleles from sequencing errors as allele frequency decreases and sequencing error rate increases. On the other hand, the EM method shows greater superiority since it is specifically designed for the purpose. However, when the sequencing error rate is very low, for example, less than one out of 2000 and *f *≥ 1%, the simple naive estimation method works reasonably well.

**Table 1:** Comparison of ${\hat{f}}_{\mathrm{em}}$ and ${\hat{f}}_{\mathrm{avg}}$ in terms of mean squared error

	*α *= 0.05%		*α *= 0.1%		*α *= 0.5%		*α *= 1%	
	
	MSE	Cg	MSE	Cg	MSE	Cg	MSE	Cg
*f *= 0.1%	0	0	0	0	0	0	0	0
*f *= 0.5%	9	5	4	3	0	0	0	0
*f *= 1%	13	10	9	7	0	0	0	0
*f *= 5%	17	16	17	16	12	12	7	7

Number of scenarios where MSE_em _> MSE_avg _or Cg_em _> Cg_avg _out of 18 total scenarios for each cell.

**Figure 1:**
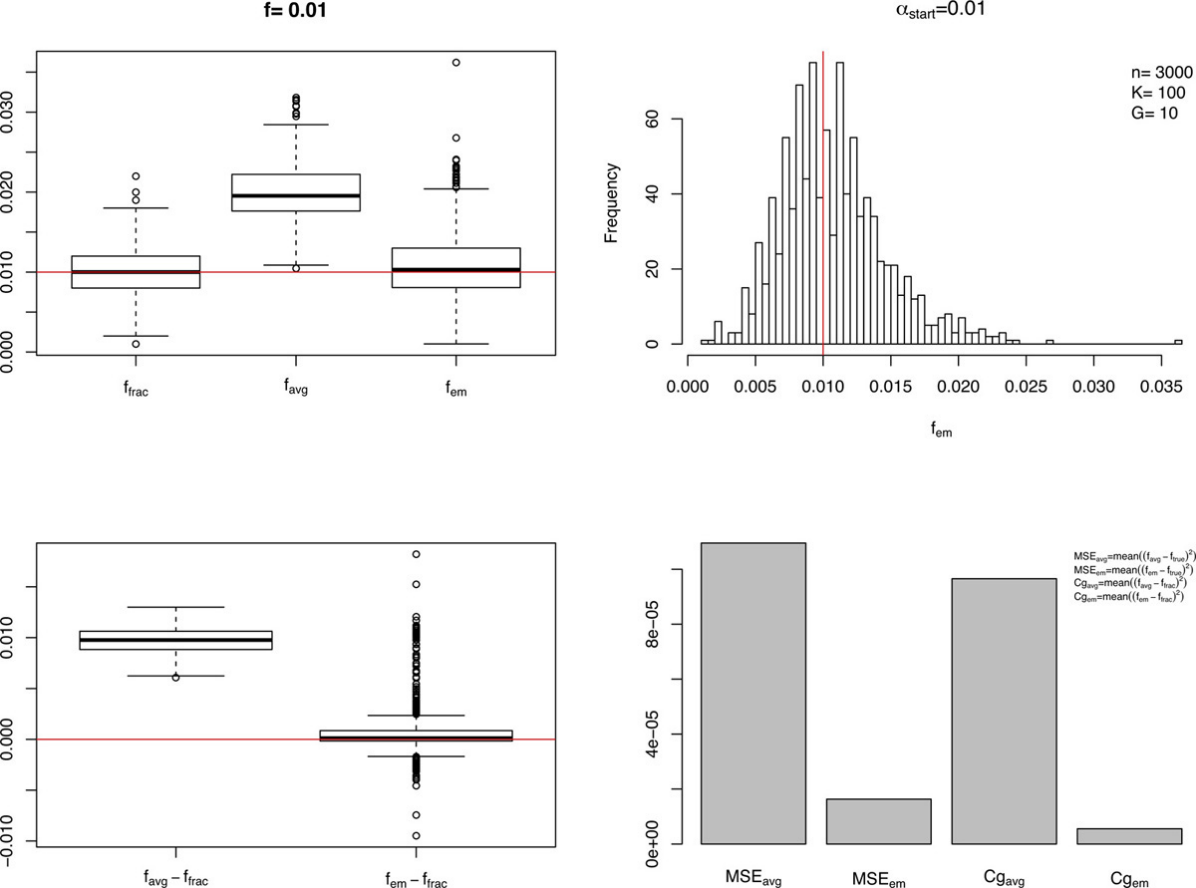
**Comparison of ${\hat{f}}_{\mathrm{em}}$ and ${\hat{f}}_{\mathrm{avg}}$**. An example for the comparison of performances between ${\hat{f}}_{\text{em}}$ and ${\hat{f}}_{\text{avg}}$, where *f *= 1%, *α*_start _= 1%, *n *= 3000, *K *= 100, and *G *= 10. The two box plots on the left are a comparison between ${\hat{f}}_{\text{avg}}$ and ${\hat{f}}_{\text{em}}$ as an estimate of *f *and ${\hat{f}}_{\mathrm{frac}}$, where ${\hat{f}}_{\text{avg}}$ shows a considerable tendency of over-estimation compared to ${\hat{f}}_{\text{em}}$. The upper right histogram shows how ${\hat{f}}_{\text{em}}$ deviates from *f *in 1000 simulations, which appears to be roughly unbiasedly distributed around *f*. The lower right bar plot is a summary plot of the MSE for these two methods as estimates of *f *and *f*_frac_, showing superiority of ${\hat{f}}_{\text{em}}$ both as an estimator of *f *and of *f*_frac_, in terms of MSE and Cg.

Figure 1 gives an example of a common pooled sequencing setting of *α*_start _= 1%, *n *= 3000, *K *= 100, *G *= 10, and a minor allele frequency of *f *= 1%. The upper left panel shows that ${\hat{f}}_{\text{avg}}$ suffers from an evident over-estimation of both *f *and *f*_frac_, while ${\hat{f}}_{\text{em}}$ appears to be an unbiased estimate of *f*. The upper right panel shows the histogram of ${\hat{f}}_{\text{em}}$ over 1000 simulations. The lower left panel shows the box plot of ${\hat{f}}_{\text{avg}}-f_{\text{frac}}$ and ${\hat{f}}_{\text{em}}-f_{\text{frac}}$, respectively. It shows that ${\hat{f}}_{\text{em}}-f_{\text{frac}}$ centers around 0, which suggests that the variance of *f*_frac _might be responsible for the majority of the variance of ${\hat{f}}_{\text{em}}$. The bar plot of the MSE for both ${\hat{f}}_{\text{avg}}$ and ${\hat{f}}_{\text{em}}$ as estimates of *f *and *f*_frac _in the lower right panel quantitatively demonstrates the superiority of ${\hat{f}}_{\text{em}}$ over ${\hat{f}}_{\text{avg}}$ in terms of their mean squared errors.

#### The relative errors of ${\hat{f}}_{\mathrm{avg}}$ and ${\hat{f}}_{\mathrm{em}}$ in estimating minor allele frequency *f*

We measure the bias of an estimator by the relative error (RE) defined as $RE=100\times |\hat{f}-f|/f$, where $\hat{f}$ is the mean of the estimates of *f *across all replications. The log values of the RE of all 288 simulations for both ${\hat{f}}_{\text{avg}}$ and ${\hat{f}}_{\text{em}}$ are given in Figure S1 of the additional file 1. The figure shows that the number of reads *n *in each pool, the number of chromosomes *K *and the number of pools *G *have little effect on the RE of ${\hat{f}}_{\text{avg}}$, while the allele frequency *f *and the sequencing error rate *α *play a dominant role in affecting RE. To further explore their effects, we demonstrate the average effects of *f *and *α *on the RE by computing the average RE based on the values of *f *and *α *across all different (*n*, *G*, *K*) in Table 2. It is interesting to observe that fixing *α*, the RE of ${\hat{f}}_{\text{avg}}$ decreases linearly with *f *; while fixing *f*, the RE of ${\hat{f}}_{\text{avg}}$ increases linearly with *α*. Thus, ${\hat{f}}_{\text{avg}}$ suffers most severely in the case of rare polymorphisms and high sequencing error rate. It can be seen from Table 2 that the RE of ${\hat{f}}_{\text{em}}$ in estimating minor allele frequency *f *is significantly lower compared to that of ${\hat{f}}_{\text{avg}}$ for rare polymorphisms at *f *≤ 1%.

**Table 2:** Comparison of ${\hat{f}}_{\mathrm{em}}$ and ${\hat{f}}_{\mathrm{avg}}$ in terms of average relative error

	*α *= 0.05%		*α *= 0.1%		*α *= 0.5%		*α *= 1%	
	
*f*	$RE_{{\hat{f}}_{\mathrm{avg}}}$	$RE_{{\hat{f}}_{\mathrm{em}}}$	$RE_{{\hat{f}}_{\mathrm{avg}}}$	$RE_{{\hat{f}}_{\mathrm{em}}}$	$RE_{{\hat{f}}_{\mathrm{avg}}}$	$RE_{{\hat{f}}_{\mathrm{em}}}$	$RE_{{\hat{f}}_{\mathrm{avg}}}$	$RE_{{\hat{f}}_{\mathrm{em}}}$
0.1%	52.0	9.4	102.0	15.6	502.0	72.0	1000.0	146.0
0.5%	10.3	4.0	20.2	4.9	99.5	13.3	199.0	26.5
1%	5.0	3.3	10.0	3.7	49.2	5.7	98.3	9.5
5%	1.0	5.4	1.9	6.0	9.1	6.7	18.1	6.3

The average RE of ${\hat{f}}_{\text{avg}}$ and ${\hat{f}}_{\text{em}}$ for different values of minor allele frequency *f *and sequencing error rate *α*.

Next we present our results for the effects of (*K*, *G*, *n*, *f*, *α*) on the estimation accuracy of minor allele frequency *f *using the EM algorithm. We note that the estimation of *β *= *f*(0*|*1) is highly unreliable (data not shown). This phenomenon can be explained as follows. When minor allele frequency *f *is low, the expected number of chromosomes having the minor allele in each pool is also low. When the number of pools *G *is small, the estimation of *β *can be difficult with a small number of chromosomes carrying the minor allele. Thus, we do not show detailed results on the estimation of *β*. Despite the fact that *β *cannot be reliably estimated, the other two parameters *f *and *α *can be reliably estimated using the EM approach.

#### The effects of minor allele frequency f and sequencing error rate α on the estimation accuracy of ${\hat{f}}_{\mathrm{em}}$

To study the effects of minor allele frequency *f *and sequencing error rate *α *on the estimation accuracy of ${\hat{f}}_{\text{em}}$, as an estimator of both *f *and *f*_frac_, we fix (*K*, *G*, *n*) = (100, 10, 3000). The histograms of ${\hat{f}}_{\text{em}}$ under each combinations of *f *and *α *are shown in Figure S2 of the additional file 1. We observe that ${\hat{f}}_{\text{em}}$ is roughly unbiasedly distributed around *f*, but the variance of ${\hat{f}}_{\text{em}}$ as an estimator of *f *is relatively large. The source of this variance, however, is largely due to the variance of *f*_frac_, rather than the algorithm itself, as shown in Figure S3 of the additional file 1, where the histograms of ${\hat{f}}_{\text{em}}-f_{\text{frac}}$ is tightly distributed around 0, with the majority of the variance shown in Figure S2 of the additional file 1 disappeared. This is an explicit evidence that the variance of ${\hat{f}}_{\text{em}}$ consists mostly of the variance of *f*_frac_. Thus, ${\hat{f}}_{\text{em}}$ might serve better as an estimator of *f*_frac _than of *f*. We also observe as a general trend that ${\hat{f}}_{\text{em}}$ appears to be a roughly unbiased estimator for both *f *and *f*_frac_, and its variance appears to be affected less by *α *but significantly by *f*. This observation is also confirmed in Table 3 where MSE's and Cg's for different combinations of *f *and *α *are shown.

**Table 3:** ${\hat{f}}_{\mathrm{em}}$ as an estimator of *f *or *f*_frac_

	*α *= 0.05%		*α *= 0.1%		*α *= 0.5%		*α *= 1%	
	
*f*	MSE	Cg	MSE	Cg	MSE	Cg	MSE	Cg
0.1%	9.8e-7	4.3e-8	9.7e-7	1.2e-7	3.2e-6	2.8e-6	1.1e-5	1.1e-5
0.5%	5.5e-6	1.9e-7	5.4e-6	1.9e-7	6.7e-6	1.5e-6	1.0e-5	5.8e-6
1%	1.2e-5	8.6e-7	1.2e-5	9.6e-7	1.3e-5	2.3e-6	1.6e-5	5.6e-6
5%	5.5e-5	1.3e-5	5.9e-5	1.7e-5	8.3e-5	4.3e-5	1.0e-4	7.0e-5

The mean squared errors (MSE) and Cg's of ${\hat{f}}_{\text{em}}$ as an estimator for *f *or *f*_frac _for various combinations of *f *and *α*, (*K*, *G*, *n*) = (100, 10, 3000).

To reduce the effect of a few outliers of ${\hat{f}}_{\text{em}}$ on the MSE and Cg calculation, we also modified the definitions of MSE and Cg by removing the top and bottom *κ*% of its values and recalculate the values of MSE and Cg. The results on the modified measures are presented in additional file 1 and the qualitative results on the performance of ${\hat{f}}_{\text{em}}$ continue to hold.

We also studied the effects of (*K*, *G*, *n*) on the estimation accuracy of ${\hat{f}}_{\text{em}}$ and the details of the simulation results are given in additional file 1. It was observed that the accuracy increases with *G *and *n *as expected. However, the accuracy decreases with the number of individuals *K *in each pool.

### Results on the power of SNP calling using the likelihood ratio test

**Figure 2:**
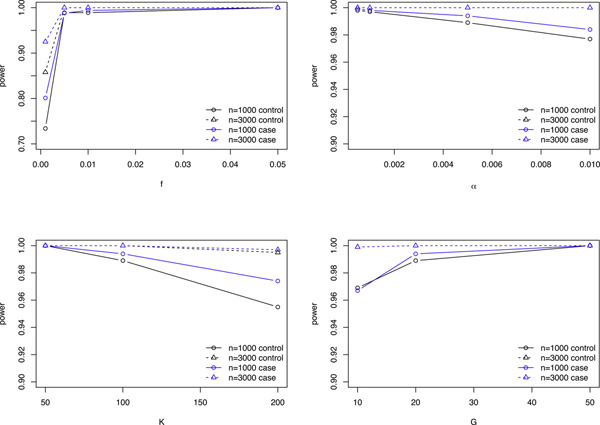
**Power of SNP detection**. The power of detecting true SNPs at a type I error of 0.05, varying one parameter at a time while fixing all other parameters at default values. Default: (*K*, *G*, *f*, *α*) = (100, 20, 1%, 0.5%, 1.2).

We next study the effects of (*K*, *G*, *n*, *f*, *α*) on the power of SNP detection using the likelihood ratio approach for the case and the control samples, respectively. The number of reads in each pool (*n*) is set at either 1000 or 3000 as in the above simulations. We start from default values of the parameters (*K*, *G*, *n*, *f*, *α*) = (100, 20, 1.2, 1%, 0.5%). Then we change one of these parameters and keep all the other parameters at default. Figure 2 shows the results for such a study and the results for using case and control samples together are given in the additional file 1 in Figure S8.

It can be seen from Figure 2 that at a type I error rate of 0.05, the power is consistently well above 0.9 in all scenarios demonstrated here except for the extremely rare variant case of *f *= 0.1%. The power tends to increase with the minor allele frequency or the number of pools, while it decreases with sequencing error rate or number of individuals in each pool. The power also increases with the number of reads in each pool. We observe that the power using the case samples is slightly higher than that using the control samples. This observation can be explained by the fact that the frequency of the minor allele in the cases is higher than that in the controls, resulting in higher power of SNP detection.

### Results on the power of association studies using the likelihood ratio test

**Figure 3:**
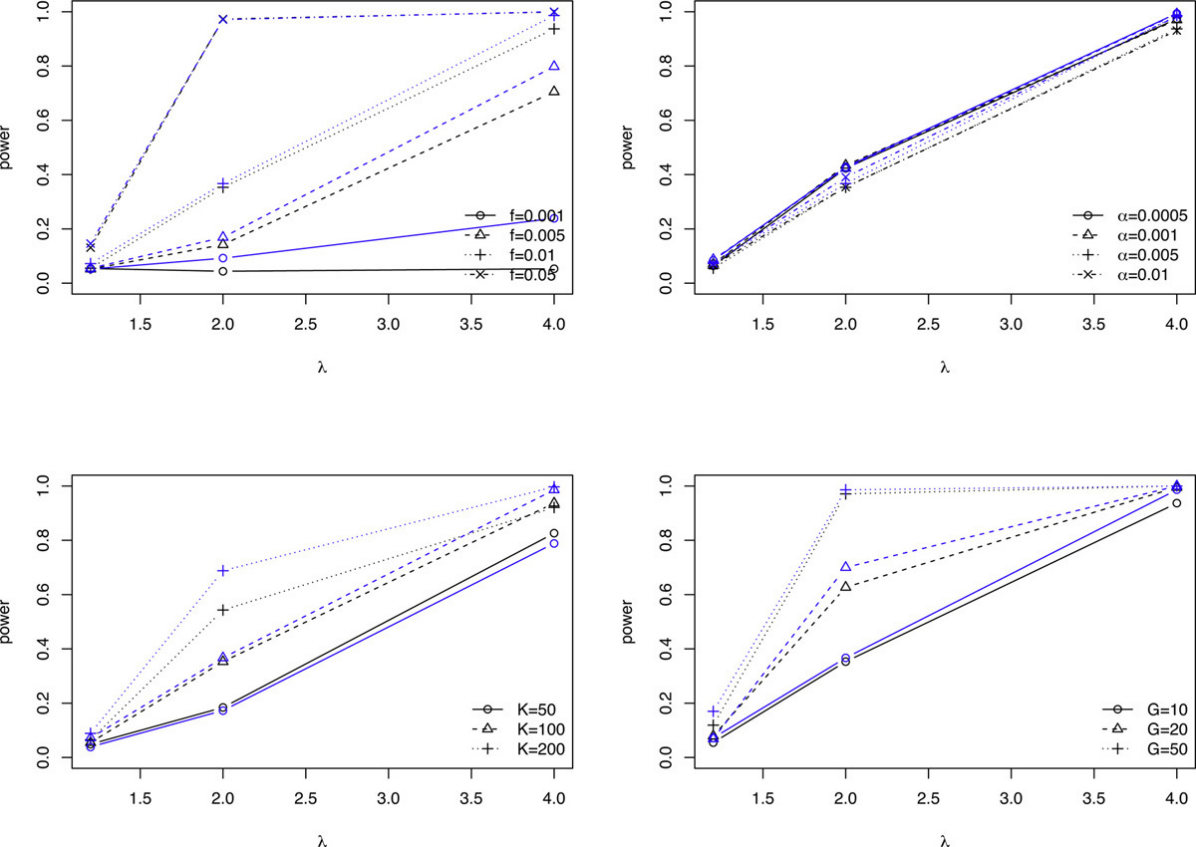
**Power of association**. The power of detecting associated SNPs at a type I error of 0.05. Each subplot displays the effect of one parameter and the number of reads *n *(black indicates *n *= 1000, and blue indicates *n *= 3000) on the power of the test, while fixing all other parameters at default values. Default: (*K*, *G*, *f*, *α*) = (100, 10, 1%, 0.5%).

We also study the effects of (*K*, *G*, *n*, *f*, *α*) on the power of detecting associations between SNPs and phenotypes using the likelihood ratio approach for the case and control samples together. The parameter setting is similar to that in the above subsection except that here we also let the relative risk *λ *to be 1.2, 2, and 4, respectively. Figure 3 shows how each parameter affects the power of detecting the association.

It can be seen from Figure 3 that at a type I error rate of 0.05, the power increases with *λ *and approaches 1 as *λ *goes up to 4, which happens in all scenarios demonstrated here except for the extremely rare variant scenario of *f *= 0.1%. The power increases with allele frequency, pool size or number of pools, while it seems robust with respect to changes in sequencing error rate.

### Results on the analysis of the type 1 diabetes data in [8]

#### Allele frequency estimation and SNP calling in the control samples

We apply our approaches to analyze the pooled sequencing data in [8]. First, we conduct SNP calling using both our method EM-SNP and SNVer (parameter setting -bq 20 -a 0 -f 0 -p 1 -t 0) [11], a program that has been shown to outperform several other programs for SNP calling including CRISP, SAMtools, and GATK. Unlike many previous programs calling variants as SNPs or not, SNVer ranks variants according to their potential of being true SNPs using the p-value defined in the program. As a likelihood ratio test, EM-SNP can also rank the variants by the magnitude of the likelihood ratio. The estimated allele frequency spectrum of the top 100 called SNPs by either EM-SNP or SNVer is given in Figure S9 of the additional file 1. Note that 5 variants have dominant non-reference alleles and they are excluded from both lists for a fair comparison. In the SNVer list, we also exclude the variants that are removed in the preprocessing stage of EM-SNP. Both frequency spectrums of the variants called by EM-SNP and SNVer tend to concentrate on the lower frequency range.

#### Evaluation of the SNP calling results

**Figure 4:**
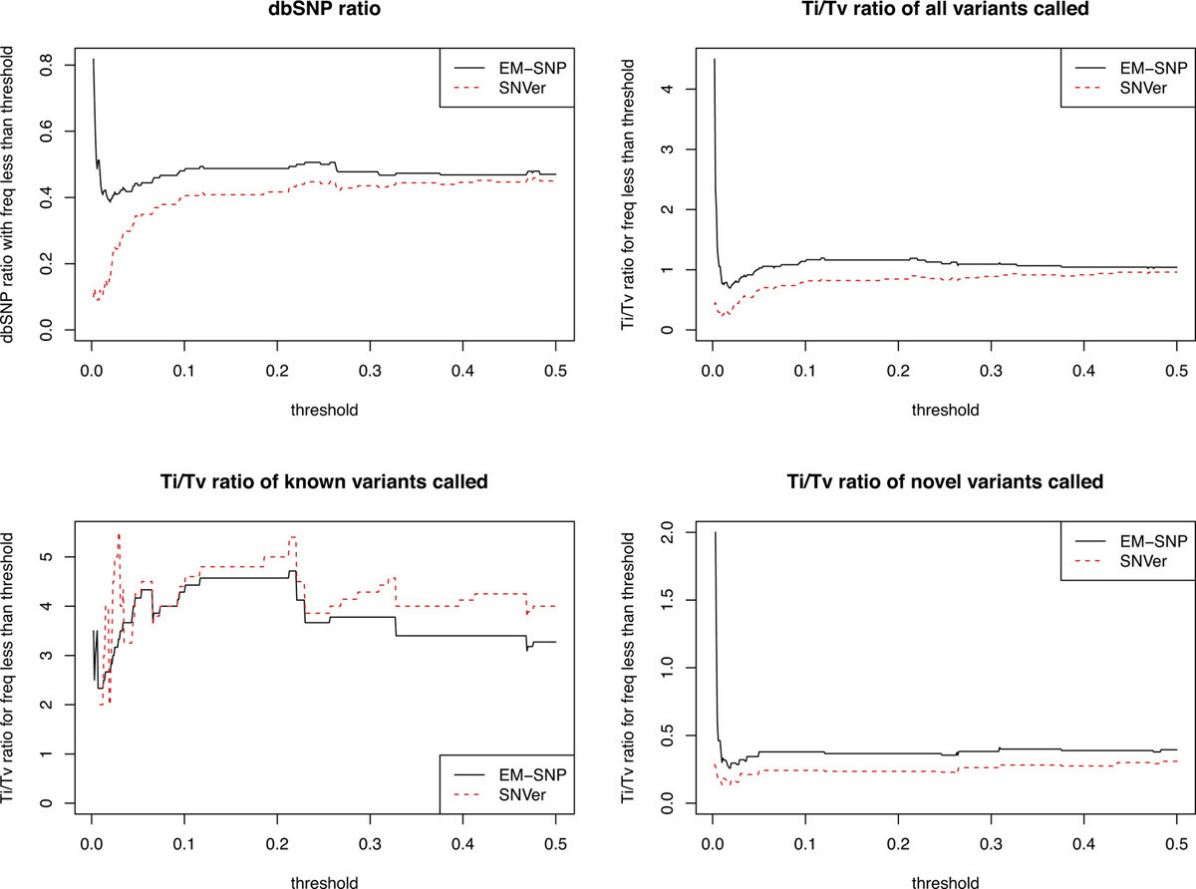
**dbSNP ratio and Ti/Tv ratio**. dbSNP ratio and Ti/Tv (transition/transversion) ratio of the top 100 variants called by EM-SNP and SNVer, whose minor allele frequencies are less than the corresponding threshold labeled by the x-axis.

A standard approach to evaluate the effectiveness of a SNP calling method is to compare the fraction of dbSNPs [24] among the top ranked SNPs, defined as the dbSNP ratio. The rationale is that if a SNP calling method is reasonable, it should be able to detect the SNPs that are already in the dbSNP database because these SNPs have been reported in previous studies. Therefore, a higher dbSNP ratio indicates potentially better performance of the SNP calling method. Figure 4 shows the cumulative dbSNP ratio of the top 100 called variants whose minor allele frequencies are less than a threshold. To further demonstrate the effect of minor allele frequency on the performance of EM-SNP and SNVer, we also show the dbSNP ratio in different windows of minor allele frequencies in Figure S10 of the additional file 1.

In terms of the dbSNP ratio for the top 100 called variants, EM-SNP consistently outperforms SNVer under all allele frequency thresholds, and EM-SNP displays significant superiority especially for low frequency variants. In Table S7 of the additional file 1, we give an example of the dbSNP ratio among the top 100 SNP calls with *f*_em _*≤ *0.2% for the two methods. Using a total of 480 control samples, EM-SNP identifies variants with minor allele frequency less than 0.2% with a high dbSNP ratio, which serves as an evidence of its superior performance in rare variant scenarios. On the other hand, the upper left panel of Figure S10 in the additional file 1 shows that the relative performance of EM-SNP and SNVer based on dbSNP ratio depends on minor allele frequency. EM-SNP detects more rare variants and has higher dbSNP ratio at minor allele frequency less than 1%. Whereas this relative performance of EM-SNP and SNVer is reversed for minor allele frequency above 1%. Thus, EM-SNP is most useful in detecting rare variants.

Another criterion to evaluate SNP calls is the transition-transversion (Ti/Tv) ratio. It is well known that transitions are much more frequent than transversions in evolution, and the number of transitions over the number of transversions, referred to as Ti/Tv ratio, in known SNPs is expected to be between 2 and 4 [25]. Figure 4 shows that EM-SNP gives a consistently higher Ti/Tv ratio throughout the entire allele frequency range for both the whole set of called variants and the novel set. For the known variants, the Ti/Tv ratio trends of the two methods are similar to each other. Table S8 in the additional file 1 gives an example of the Ti/Tv ratio among the top 100 SNP calls with *f*_EM _< 0.2% by EM-SNP and SNVer. The effect of minor allele frequency on the relative performance of EM-SNP and SNVer in terms of Ti/Tv ratio is similar to that in terms of dbSNP ratio (Figure S10 in the additional file 1).

We also consider the top 150 ranked SNPs and the corresponding figures and tables are shown as Figure S11-S12 and Tables S7-S8 in the additional file 1. The qualitative conclusions are the same.

### Identifying SNPs associated with type 1 diabetes

We then study the association of the identified variants with type 1 diabetes (T1D). We first look at the common SNPs with estimated minor allele frequencies above 1% in the controls as in [8] and want to see if we can identify the common SNPs associated with T1D. With the estimated allele frequencies, we can estimate the numbers of minor alleles in the controls and in the cases separately. Based on the estimated counts, we obtain a preliminary p-value based on the Fisher's exact test as in [8]. However, the p-value obtained this way is not accurate as it does not consider the variation in estimating the allele frequencies using the EM algorithm. Therefore, we then use the likelihood ratio statistic defined in equation 8 to calculate another p-value that is given in the last column in Table 4, where we only list SNPs with preliminary p-value less than 10^-5^. The p-value obtained through the likelihood ratio test reflects the true p-value better because it takes the variation in estimating the allele frequency into account.

**Table 4:** Association results

SNP	Gene	${\hat{f}}_{0}$	*n*_0_	${\hat{f}}_{1}$	*n*_1_	Fisher's p-value	EM p-value
rs3184504	SH2B3	0.52	499	0.41	394	1.9e-6	8.4e-7
rs7076103	IL2RA	0.19	178	0.10	93	4.5e-8	2.7e-7
rs2476601	PTPN22	0.09	86	0.16	151	8.1e-6	9.2e-6

Testing for association between common SNPs $\left({\hat{f}}_{0}\geq 1\%\right)$ and T1D with Fisher's preliminary p-value at most *e*-5. In the table, ${\hat{f}}_{0}$ and ${\hat{f}}_{1}$ are the estimated minor allele frequencies using the EM algorithm in the controls and cases, respectively, and $n_{i}=\left\lfloor 960\times {\hat{f}}_{i}\right\rfloor$, *i *= 0, 1. The Fisher's p-value is the preliminary p-value using the Fisher's exact test as in [8] and the EM p-value is the p-value calculated based on the likelihood ratio statistic in equation 8.

The SNP rs3184504 residing within gene SH2B3 has an EM p-value of 8.4*e *- 7. This SNP was also found to be associated with a preliminary p-value of 5*e *- 7 in the original study [8] and the corresponding gene was previously identified to be associated with T1D. The fractions of observed minor alleles in controls and cases in the original study were 53% and 41.5%, respectively, and our mapping results are consistent with the estimates. The EM estimated minor allele frequencies in the controls and cases are 52% and 41%, which are slightly smaller than the observed values since we took sequencing errors into account. The SNP rs7076103 within the gene IL2RA has a preliminary Fisher's p-value of 4.5e-8 and an EM p-value of 2.7e-07. This SNP was not reported to be associated with any phenotypes according to the catalog of GWAS studies (http://www.genome.gov/gwastudies/) to date. Nevertheless, other SNPs within the IL2RA gene were found to be associated with T1D by Cooper et al. [26] before the publication of [8] and by two other studies [27,28] after the publication of [8] using different approaches. Barrett et al. [27] used genome-wide association studies giving a p-value of 1.0e-13 while Huang et al. [28] used imputation of the genotypes based on the 1000 genome projects yielding a p-value of 5e-9. These new studies support our significant result on the association of rs7076103 with T1D. The estimated minor allele frequencies in the controls and cases were 18.8% and 14.8% in [8] giving a p-value of 0.02 which is not significant after adjusting for multiple testing. This example shows that even for common polymorphisms, the EM approach can help to find likely associations that the naive approach can not. The SNP rs2476601 was found to be associated with T1D in several studies before the publication of [8] and was confirmed in a recent study in [29] that was published after the publication of [8]. All these studies support our results for the association of common polymorphisms with T1D.

For rare polymorphisms $\left({\hat{f}}_{0}<\text{1}\%\right)$ within the controls, we first use the naive approach described above to obtain a preliminary Fisher's p-value for every SNP. Due to the low minor allele frequencies of the rare variants, none of the p-values is smaller than 0.001. We did not pursue the association of individual variants with T1D further.

## Discussion

In this paper, we developed an EM algorithm based unified approach for minor allele frequency estimation, SNP calling and association studies, applicable to pooled sequencing data where genetic materials of multiple individuals are pooled together. This study differs from previous studies in that we estimate sequencing error rate for each position while previous studies generally assume a pre-specified sequencing error rate across all sequenced regions. Since sequencing error rate depends on the genomic context, it is essential that the sequencing error rate be estimated specifically for different loci. In a pooling design without tagging, the origin of the reads is not known, and it is impossible to obtain the individual genotypes from the pooled data. Therefore, we modelled the pooled sequencing data as a "missing value" problem and designed an EM algorithm to estimate the minor allele frequency and sequencing error rate.

We first studied the effects of minor allele frequency, sequencing error rate, number of pools, number of individuals in each pool, and the sequencing depth in each pool, on the estimation accuracy of the minor allele frequency. It was shown that the naive approach, which estimates the minor allele frequency by the fraction of observed minor alleles in the reads, can significantly over-estimate the true minor allele frequency, and that the effect is most severe for rare variants. The EM based algorithm, on the other hand, can estimate the minor allele frequency in a relatively unbiased manner. Although the variation of this estimation seems to be relatively large, a major part of the variation comes from the sampling of individuals from the population rather than the algorithm itself. We also show that the estimation accuracy of the EM algorithm increases with the number of pools and sequence depth as expected. However, the estimation accuracy decreases with the number of individuals in each pool, most likely because a more extensive pooling induces greater loss of information. Secondly, we used a likelihood ratio statistic based on the estimated parameters from EM to call SNPs. With the real data from [8], in terms of the dbSNP ratio, we showed that EM-SNP outperforms SNVer for rare variants with minor allele frequency less than 1%. We also showed that the transition/transversion ratio of the called SNPs for rare variants based on EM-SNP is higher than that of the called SNPs by SNVer. These two independent pieces of evidence demonstrate that EM-SNP is superior to SNVer in the discovery of rare variants. However, the extent of this advantage decreases as minor allele frequency increases due to the tradeoff between EM-SNP's bias adjustment for the estimation of minor allele frequencies and extra variation introduced in the EM algorithm. Finally, we applied our approach to reanalyze the case-control data from [8] and showed that we can find the associated common SNPs. Unfortunately we did not find any significantly associated rare variants. One possible explanation is that the power of finding rare variants associated with complex traits is generally low as a consequence of the low frequencies of minor alleles.

We made several simplifying assumptions in our study. First and foremost, we did not consider errors introduced by mapping the reads to the reference genome. The mapping of Roche 454 data still has many challenges, in particular, in regions around homopolymers, and further development of algorithms for mapping is needed. Secondly, although we assumed that the amount of genetic materials from each individual is the same for each pool, this assumption can be violated. To overcome this problem, one approach would assume that the fractions of genetic materials from individuals follow a Dirichlet distribution [17]. Thirdly, the called SNPs by EMSNP still have many false positives since the Ti/Tv ratio for the called novel SNPs is still low compared to the known SNPs. Further improvements in SNP calling are needed. Finally, the computational speed of the EM based approach can be relatively slow, and the method cannot be applied to whole genome association studies although this is not a problem for targeted sequencing studies as in [8]. These are the topics for future research.

## Software

Software can be downloaded from http://www-rcf.usc.edu/~fsun/Programs/EM-SNP/EM-SNP.html.

## Competing interests

The authors declare that they have no competing interests.

## Authors' contributions

Both authors participated in the development of methodology, simulations, real data application, revisions, and manuscript preparation. Both authors read and approved the final manuscript.

## Declarations

The publication costs for this article were funded by US NIH 1 U01 HL108634.

This article has been published as part of *BMC Genomics *Volume 14 Supplement 1, 2013: Selected articles from the Eleventh Asia Pacific Bioinformatics Conference (APBC 2013): Genomics. The full contents of the supplement are available online at http://www.biomedcentral.com/bmcgenomics/supplements/14/S1.

## Supplementary Material

Additional file 1 **Supplementary materials**. Supplementary methods and results. Click here for file

## References

[B1] Yang J Benyamin B McEvoy BP Gordon S Henders AK Nyholt DR Madden PA Heath AC Martin NG Montgomery GW Goddard ME Visscher PM Common SNPs explain a large proportion of the heritability for human height Nature Genetics 2010 42 565 569 10.1038/ng.608 20562875 PMC3232052

[B2] Manolio TA Collins FS Cox NJ Goldstein DB Hindorff LA Hunter DJ McCarthy MI Ramos EM Cardon LR Chakravarti A Cho JH Guttmacher AE Kong A Kruglyak L Mardis E Rotimi CN Slatkin M Valle D Whittemore AS Boehnke M Clark AG Eichler EE Gibson G Haines JL Mackay TFC McCarroll SA Visscher PM Finding the missing heritability of complex diseases Nature 2009 461 747 753 10.1038/nature08494 19812666 PMC2831613

[B3] Nelson MR Wegmann D Ehm MG Kessner D Jean PS Verzilli C Shen J Tang Z Bacanu SA Fraser D Warren L Aponte J Zawistowski M Liu X Zhang H Zhang Y Li J Li Y Li L Woollard P Topp S Hall MD Nangle K Wang J Abecasis G Cardon LR Zöllner S Whittaker JC Chissoe SL Novembre J Mooser V An abundance of rare functional variants in 202 drug target genes sequenced in 14,002 people Science 2012 337 100 104 10.1126/science.1217876 22604722 PMC4319976

[B4] Tennessen JA Bigham AW O'Connor TD Fu W Kenny EE Gravel S McGee S Do R Liu X Jun G Kang HM Jordan D Leal SM Gabriel S Rieder MJ Abecasis G Altshuler D Nickerson DA Boerwinkle E Sunyaev S Bustamante CD Bamshad MJ Akey JM Evolution and functional impact of rare coding variation from deep sequencing of human exomes Science 2012 337 64 69 10.1126/science.1219240 22604720 PMC3708544

[B5] Benaglio P McGee TL Capelli LP Harper S Berson EL Rivolta C Next generation sequencing of pooled samples reveals new SNRNP200 mutations associated with retinitis pigmentosa Human Mutation 2011 32 E2246 2258 10.1002/humu.21485 21618346

[B6] Rivas MA Beaudoin M Gardet A Stevens C Sharma Y Zhang CK Boucher G Ripke S Ellinghaus D Burtt N Fennell T Kirby A Latiano A Goyette P Green T Halfvarson J Haritunians T Korn JM Kuruvilla F Lagacé C Neale B Lo KS Schumm P Törkvist L Dubinsky MC Brant SR Silverberg MS Duerr RH Altshuler D Gabriel S Lettre G Franke A D'Amato M McGovern DPB Cho JH Rioux JD Xavier RJ Daly MJ Deep resequencing of GWAS loci identifies independent rare variants associated with inflammatory bowel disease Nature Genetics 2011 43 1066 1073 10.1038/ng.952 21983784 PMC3378381

[B7] Out AA van Minderhout IJHM Goeman JJ Ariyurek Y Ossowski S Schneeberger K Weigel D van Galen M Taschner PEM Tops CMJ Breuning MH van Ommen GJB den Dunnen JT Devilee P Hes FJ Deep sequencing to reveal new variants in pooled DNA samples Human Mutation 2009 30 1703 1712 10.1002/humu.21122 19842214

[B8] Nejentsev S Walker N Riches D Egholm M Todd JA Rare variants of IFIH1, a gene implicated in antiviral responses, protect against type 1 diabetes Science 2009 324 387 389 10.1126/science.1167728 19264985 PMC2707798

[B9] Druley TE Vallania FLM Wegner DJ Varley KE Knowles OL Bonds JA Robison SW Doniger SW Hamvas A Cole FS Fay JC Mitra RD Quantification of rare allelic variants from pooled genomic DNA Nature Methods 2009 6 263 265 10.1038/nmeth.1307 19252504 PMC2776647

[B10] Bansal V A statistical method for the detection of variants from next-generation resequencing of DNA pools Bioinformatics 2010 26 i318 i324 10.1093/bioinformatics/btq214 20529923 PMC2881398

[B11] Wei Z Wang W Hu P Lyon GJ Hakonarson H SNVer: a statistical tool for variant calling in analysis of pooled or individual next-generation sequencing data Nucleic Acids Research 2011 39 e132 10.1093/nar/gkr599 21813454 PMC3201884

[B12] Li H Durbin R Fast and accurate long-read alignment with Burrows-Wheeler transform Bioinformatics 2010 26 589 595 10.1093/bioinformatics/btp698 20080505 PMC2828108

[B13] Altmann A Weber P Quast C Rex-Haffner M Binder EB Müller-Myhsok B vipR: variant identification in pooled DNA using R Bioinformatics 2011 27 i77 i84 10.1093/bioinformatics/btr205 21685105 PMC3117388

[B14] Kim SY Li Y Guo Y Li R Holmkvist J Hansen T Pedersen O Wang J Nielsen R Design of association studies with pooled or un-pooled next-generation sequencing data Genetic Epidemiology 2010 34 479 491 10.1002/gepi.20501 20552648 PMC5001557

[B15] Wang T Lin CY Rohan TE Ye K Resequencing of pooled DNA for detecting disease associations with rare variants Genetic Epidemiology 2010 34 492 501 10.1002/gepi.20502 20578089 PMC4096227

[B16] Lee JS Choi M Yan X Lifton RP Zhao H On optimal pooling designs to identify rare variants through massive resequencing Genetic Epidemiology 2011 35 139 147 10.1002/gepi.20561 21254222 PMC3176340

[B17] Chen X Listman JB Slack FJ Gelernter J Zhao H Biases and errors on allele frequency estimation and disease association tests of next generation sequencing of pooled samples Genetic Epidemiology 2012 (Epub ahead of print) 10.1002/gepi.21648 PMC3477622 22674656

[B18] Self SG Liang KY Asymptotic properties of maximum likelihood estimators and likelihood ratio tests under nonstandard conditions Journal of the American Statistical Association 1987 82 605 610 10.1080/01621459.1987.10478472

[B19] Quinlan AR Stewart DA Strömberg MP Marth GT Pyrobayes: an improved base caller for SNP discovery in pyrosequences Nature Methods 2008 5 179 181 10.1038/nmeth.1172 18193056

[B20] Flicek P Birney E Sense from sequence reads: methods for alignment and assembly Nature Methods 2009 6 S6 S12 10.1038/nmeth.1376 19844229

[B21] Hillier LW Marth GT Quinlan AR Dooling D Fewell G Barnett D Fox P Glasscock JI Hickenbotham M Huang W Magrini VJ Richt RJ Sander SN Stewart DA Stromberg M Tsung EF Wylie T Schedl T Wilson RK Mardis ER Whole-genome sequencing and variant discovery in C. elegans Nature Methods 2008 5 183 188 10.1038/nmeth.1179 18204455

[B22] Li H Handsaker B Wysoker A Fennell T Ruan J Homer N Marth G Abecasis G Durbin R The sequence alignment/map format and SAMtools Bioinformatics 2009 25 2078 2079 10.1093/bioinformatics/btp352 19505943 PMC2723002

[B23] Wang K Li M Hakonarson H ANNOVAR: functional annotation of genetic variants from highthroughput sequencing data Nucleic Acids Research 2010 38 e164 e164 10.1093/nar/gkq603 20601685 PMC2938201

[B24] Sherry ST Ward M Sirotkin K dbSNP--Database for single nucleotide polymorphisms and other classes of minor genetic variation Genome Research 1999 9 677 679 10447503

[B25] DePristo MA Banks E Poplin R Garimella KV Maguire JR Hartl C Philippakis AA Angel Gd Rivas MA Hanna M McKenna A Fennell TJ Kernytsky AM Sivachenko AY Cibulskis K Gabriel SB Altshuler D Daly MJ A framework for variation discovery and genotyping using next-generation DNA sequencing data Nature Genetics 2011 43 491 498 10.1038/ng.806 21478889 PMC3083463

[B26] Cooper JD Smyth DJ Smiles AM Plagnol V Walker NM Allen JE Downes K Barrett JC Healy BC Mychaleckyj JC Warram JH Todd JA Meta-analysis of genome-wide association study data identifies additional type 1 diabetes risk loci Nature Genetics 2008 40 1399 1401 10.1038/ng.249 18978792 PMC2635556

[B27] Barrett JC Clayton DG Concannon P Akolkar B Cooper JD Erlich HA Julier C Morahan G Nerup J Nierras C Plagnol V Pociot F Schuilenburg H Smyth DJ Stevens H Todd JA Walker NM Rich SS Genomewide association study and meta-analysis find that over 40 loci affect risk of type 1 diabetes Nature Genetics 2009 41 703 707 10.1038/ng.381 19430480 PMC2889014

[B28] Huang J Ellinghaus D Franke A Howie B Li Y 1000 Genomes-based imputation identifies novel and refined associations for the Wellcome Trust Case Control Consortium phase 1 Data European Journal of Human Genetics 2012 20 801 805 10.1038/ejhg.2012.3 22293688 PMC3376268

[B29] Plagnol V Howson JMM Smyth DJ Walker N Hafler JP Wallace C Stevens H Jackson L Simmonds MJ Bingley PJ Gough SC Todd JA Consortium TDG Genome-wide association analysis of autoantibody positivity in type 1 diabetes cases PLoS Genetics 2011 7 e1002216 10.1371/journal.pgen.1002216 21829393 PMC3150451

