# Supplementary material for: A unified approach for allele frequency estimation, SNP detection and association studies based on pooled sequencing data using EM algorithms
Source: BMC Genomics. 2013 Jan 21;14(Suppl 1):S1. doi: 10.1186/1471-2164-14-S1-S1 (PMC3549804; doi:10.1186/1471-2164-14-S1-S1)
Supplement: Additional file 1 — Supplementary materials. Supplementary methods and results. [file 1471-2164-14-S1-S1-S1.pdf]

Supplementary Materials for "A unified approach for allele frequency estimation, SNP detection and association studies based on pooled sequencing data using EM algorithms"

Quan Chen[1], Fengzhu Sun*[1,2]

[1]Molecular and Computational Biology Program, University of Southern California, Los Angeles, CA 90089-2910, USA
[2]TNLIST/Department of Automation, Tsinghua University, Beijing 100084, PR China

Email: Quan Chen - quanchen@usc.edu; Fengzhu Sun*- fsun@usc.edu;

*Corresponding author

Supplementary Methods

Details of simulations studies on estimating the power of association tests using EM

To study the power of testing for association between a SNP and a phenotype of interest using the method in the "Testing for associations between a SNP and a phenotype in case-control studies" subsection, we first generate case-control pooled sequencing data on loci that are not associated with the phenotype and repeat the process $R = 1000$ times. We then obtain the empirical distributions of Λ. For a given type I error γ, we take the upper γ percentile t_γ as the threshold to decide whether a locus is associated with the phenotype. Then we simulate case-control data with true association for $R = 1000$ times, and compute the fraction of times that the value of Λ is above t_γ, which is taken as an estimate of the power of association with the phenotype.

Supplementary Results

Results on the accuracy of $\hat{f}_{\text{em}}$ using the modified measures of MSE and Cg

There are a few outlier estimates that are far away from the true value. This indicates that the standard MSE criterion used in the main text may not effectively reflect the estimation accuracy of the parameters. Thus, we modify the MSE as follows. First, remove the upper κ percent and the lower κ percent of the estimated values of $\hat{f}_{\text{em}}$. Second, calculate the mean squared error of the remaining $\hat{f}_{\text{em}}$'s, denoted as $\sigma^2_m(\kappa)$, where the subscript m refers to this modified version. Here we let $\kappa = 5$ and 10 and the values of $\sigma^2_m(\kappa)$'s are presented in supplementary Tables S1 and S2, corresponding to the 16 scenarios in supplementary Figures S2 and S3.

The effects of the number of chromosomes K in a pool, the number of pools G, and the number of reads n on the estimation accuracy of $\hat{f}_{\text{em}}$

To study the effects of the number of chromosomes K in a pool and the number of pools G on the estimation accuracy of $\hat{f}_{\text{em}}$ as an estimator of f, we fix $f = 1\%, \alpha_{\text{start}} = 1\%$ and plot the histograms of $\hat{f}_{\text{em}}$ with $n = 1000$ in supplementary Figure S4. It shows that $\hat{f}_{\text{em}}$ maintains the nice property of unbiasedness around the true value f as shown in the "The effects of minor allele frequency f and sequencing error rate α on the estimation accuracy of $\hat{f}_{\text{em}}$" subsection, while $\hat{f}_{\text{em}}$ as an estimator of f displays considerable variance at the same time. However, in supplementary Figure S5 where we plot the histograms of $\hat{f}_{\text{em}} - f_{\text{frac}}$ when $n = 1000$, the variance of $\hat{f}_{\text{em}} - f_{\text{frac}}$ is significantly reduced compared to $\hat{f}_{\text{em}}$ in supplementary Figure S4. The smaller the value of K is, the more significant the reduction in variance is, which is also confirmed quantitatively in supplementary Table S3. This observation indicates that when the number of chromosomes K in each pool is relatively low, $\hat{f}_{\text{em}}$ is a fairly accurate estimate of the fraction of chromosomes f_{frac} carrying minor alleles, and the variance of $\hat{f}_{\text{em}}$ is mostly due to the variance of f_{frac}, which is an intrinsic variance of the sampling procedure from the population.

As the number of chromosomes K in each pool increases, the variance induced by the algorithm itself plays a more important role, while the variance of f_{frac} contributes less to the variance of $\hat{f}_{\text{em}}$ due to the larger sample size. In addition, supplementary Figures S4 and S5 show a consistent trend of an increase in the accuracy of $\hat{f}_{\text{em}}$ (or decrease in the MSE) as the number of pools G increases and as the number of chromosomes K in a pool decreases. This can also be seen from supplementary Table S3.

To study the effect of the number of reads n on the estimation accuracy of f_{em} for f, we also let $n = 3000$. The results are presented in supplementary Figures S6 and S7, and supplementary Tables S4 and Table S6. It can be seen that the accuracy of $\hat{f}_{\text{em}}$ increases with the number of reads in each pool.

Supplementary Figures

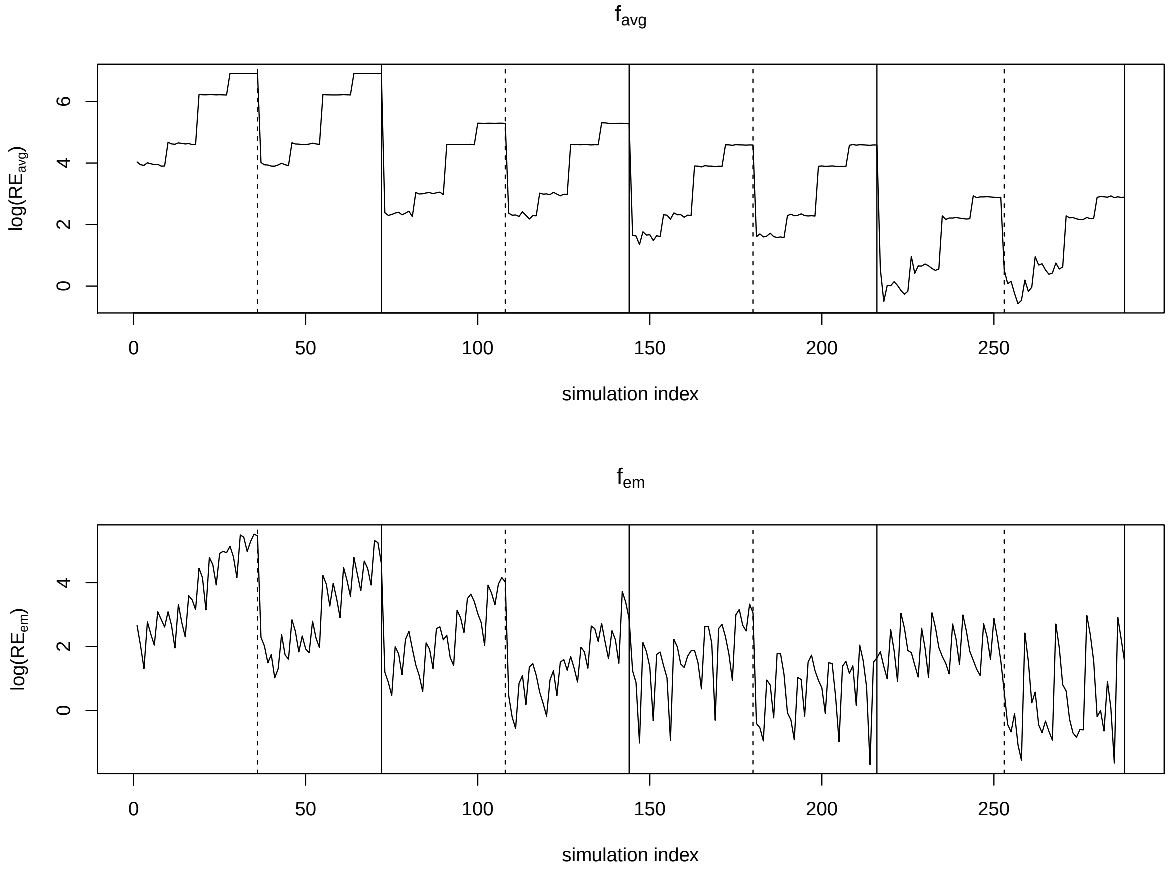

Figure S1: The log values of the relative errors (RE) of $\hat{f}_{\text{avg}}$ and $\hat{f}_{\text{em}}$ for the 288 simulations. The four sections (72 simulations each) divided by the solid lines correspond to simulations with $f = 0.1\%, 0.5\%, 1\%, 5\%$, respectively. For $\hat{f}_{\text{avg}}$ the decrease by section indicates the significant effect of f on RE. Within each section the two subsections (36 simulations each) divided by the dashed line correspond to simulations with $n = 1000, 3000$, respectively. For $\hat{f}_{\text{avg}}$ the indifference between subsections indicate no effects of n on RE. Within each subsection the four quarters (9 simulations each) correspond to simulations with $\alpha = 0.05\%, 0.1\%, 0.5\%, 1\%$, respectively. For $\hat{f}_{\text{avg}}$ the step-like shape indicates little effect of G, K on RE, compared to significant effect of α.

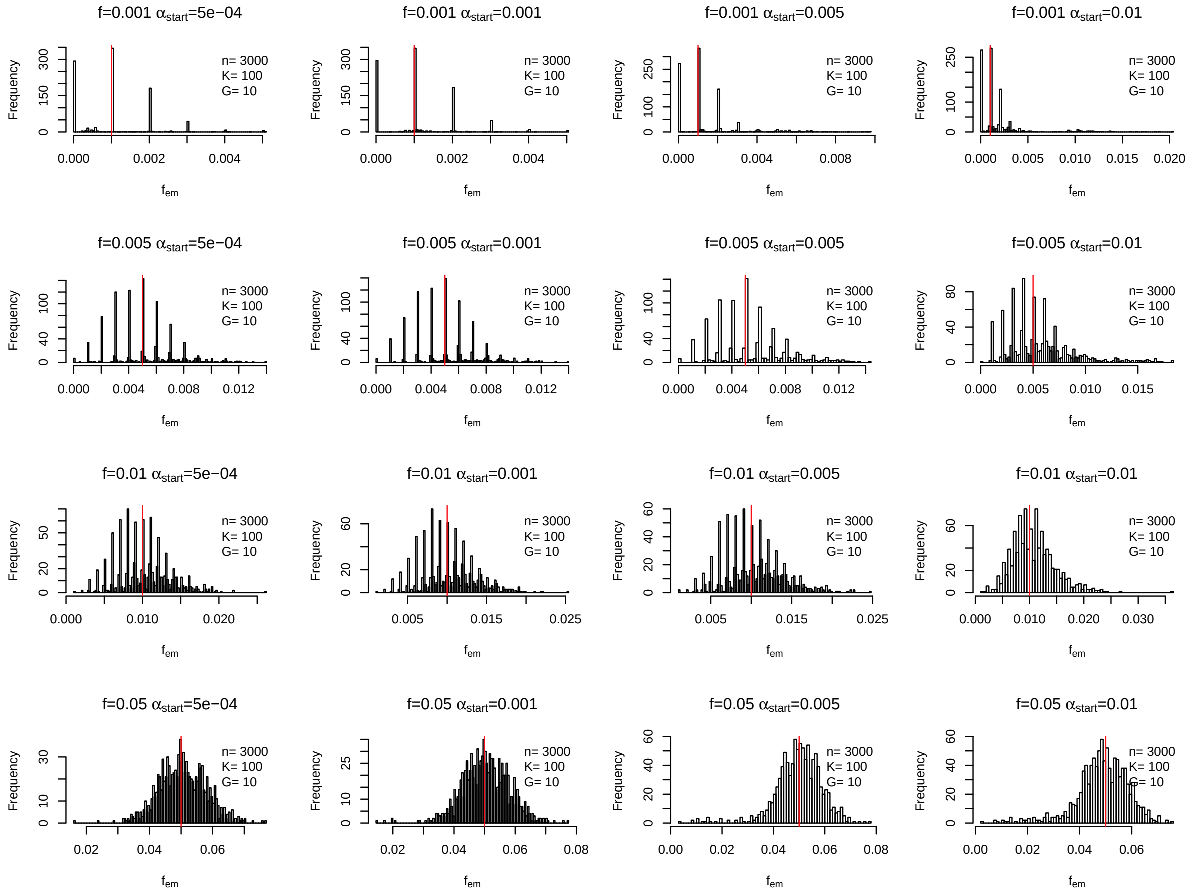

Figure S2: The effects of f and α on the accuracy of $\hat{f}_{\text{em}}$ as an estimator of f. The histograms of $\hat{f}_{\text{em}}$ under different combinations of f and α are shown, $(K, G, n) = (100, 10, 3000)$.

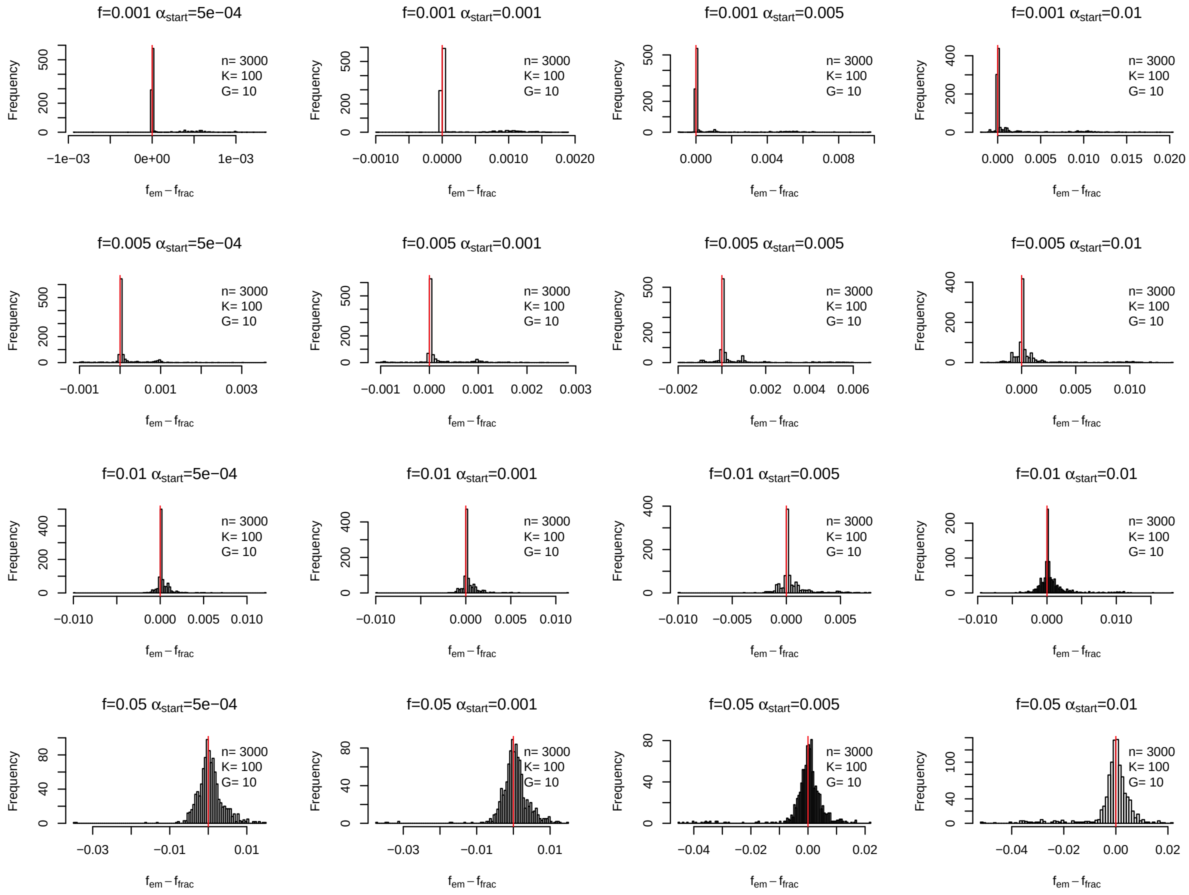

Figure S3: The effects of f and α on the accuracy of $\hat{f}_{\text{em}}$ as an estimator of f_{frac}. The histograms of $\hat{f}_{\text{em}} - f_{\text{frac}}$ under different combinations of f and α are shown, $(K, G, n) = (100, 10, 3000)$.

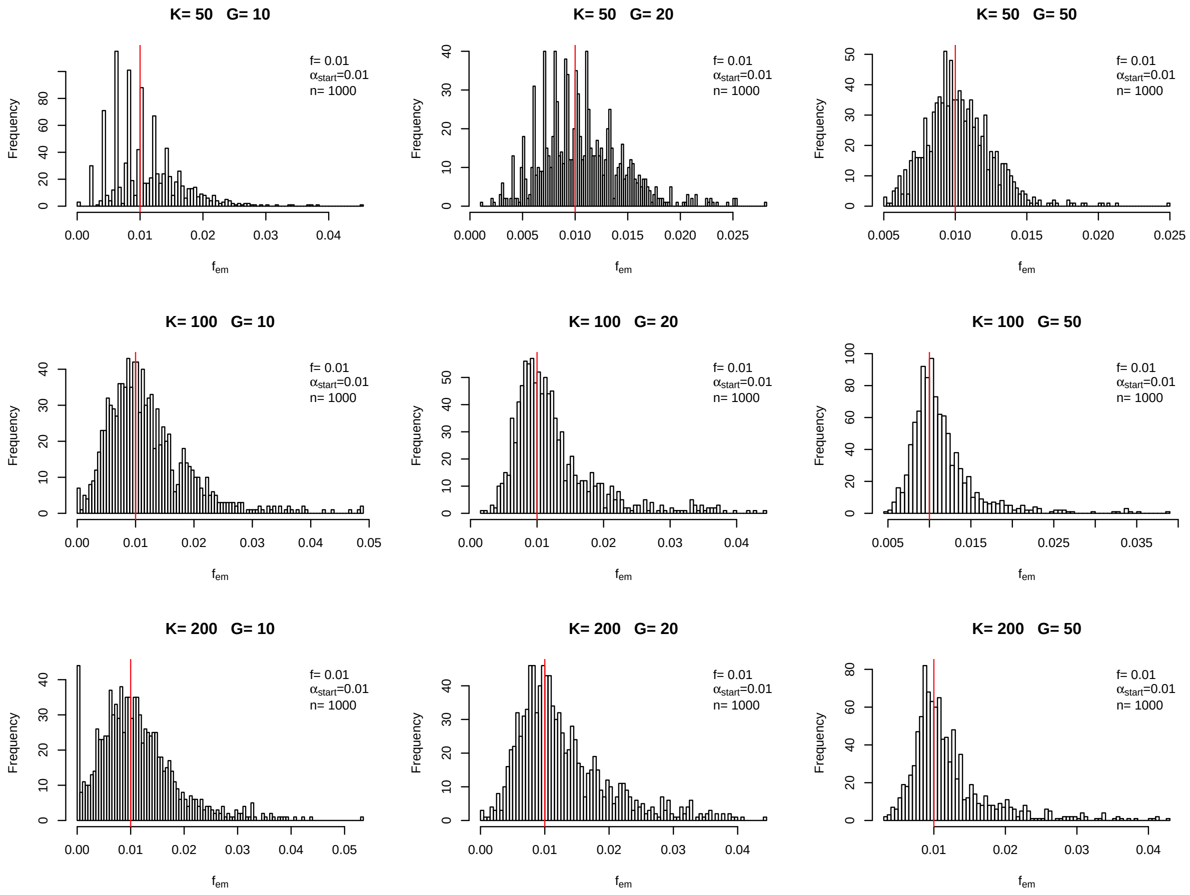

Figure S4: The effects of (K, G) on the estimation accuracy of $\hat{f}_{\text{em}}$ as an estimator of f, $(n, f, \alpha_{\text{start}}) = (1000, 0.01, 0.01)$.

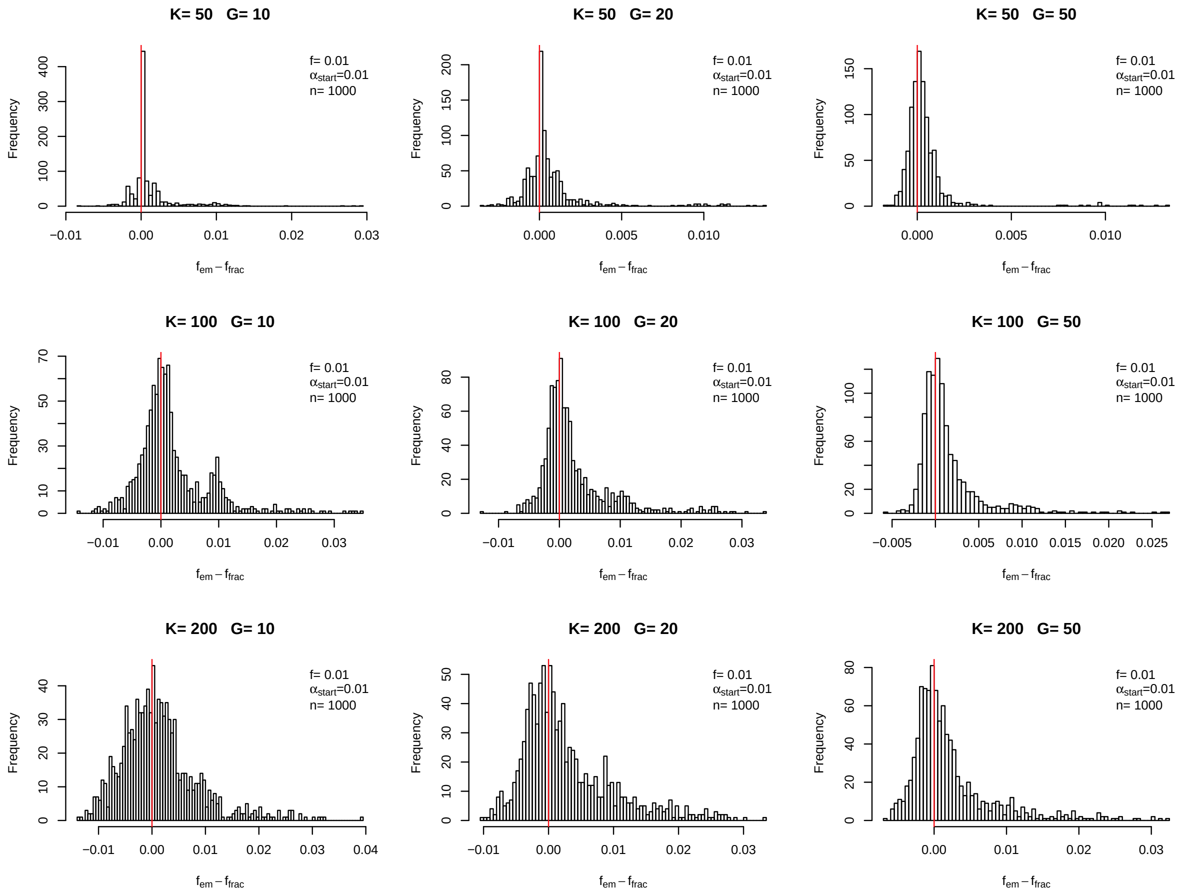

Figure S5: The effects of (K, G) on the estimation accuracy of $\hat{f}_{\text{em}}$ as an estimator of f_{frac}, $(n, f, \alpha_{\text{start}}) = (1000, 0.01, 0.01)$.

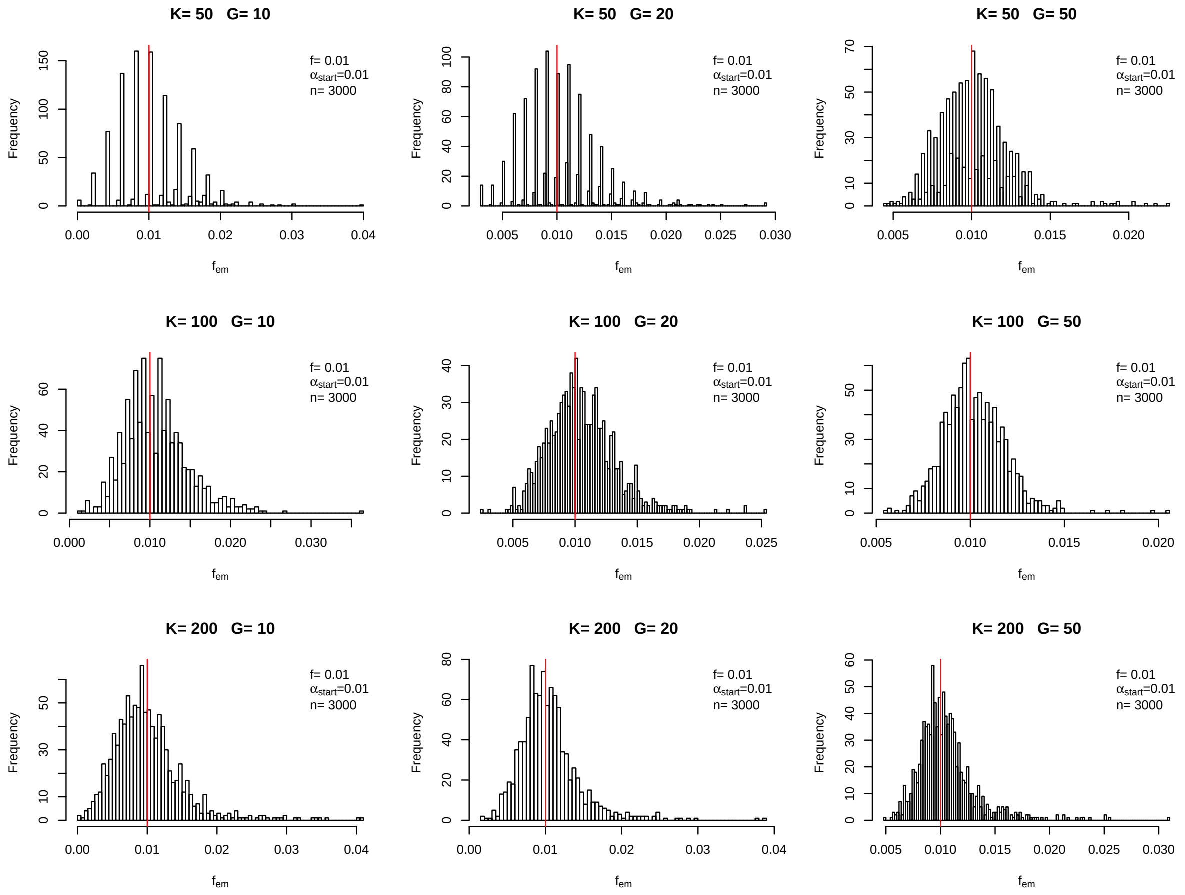

Figure S6: The effects of (K, G) on the estimation accuracy of $\hat{f}_{\text{em}}$ as an estimator of f, $(n, f, \alpha_{\text{start}}) = (3000, 0.01, 0.01)$.

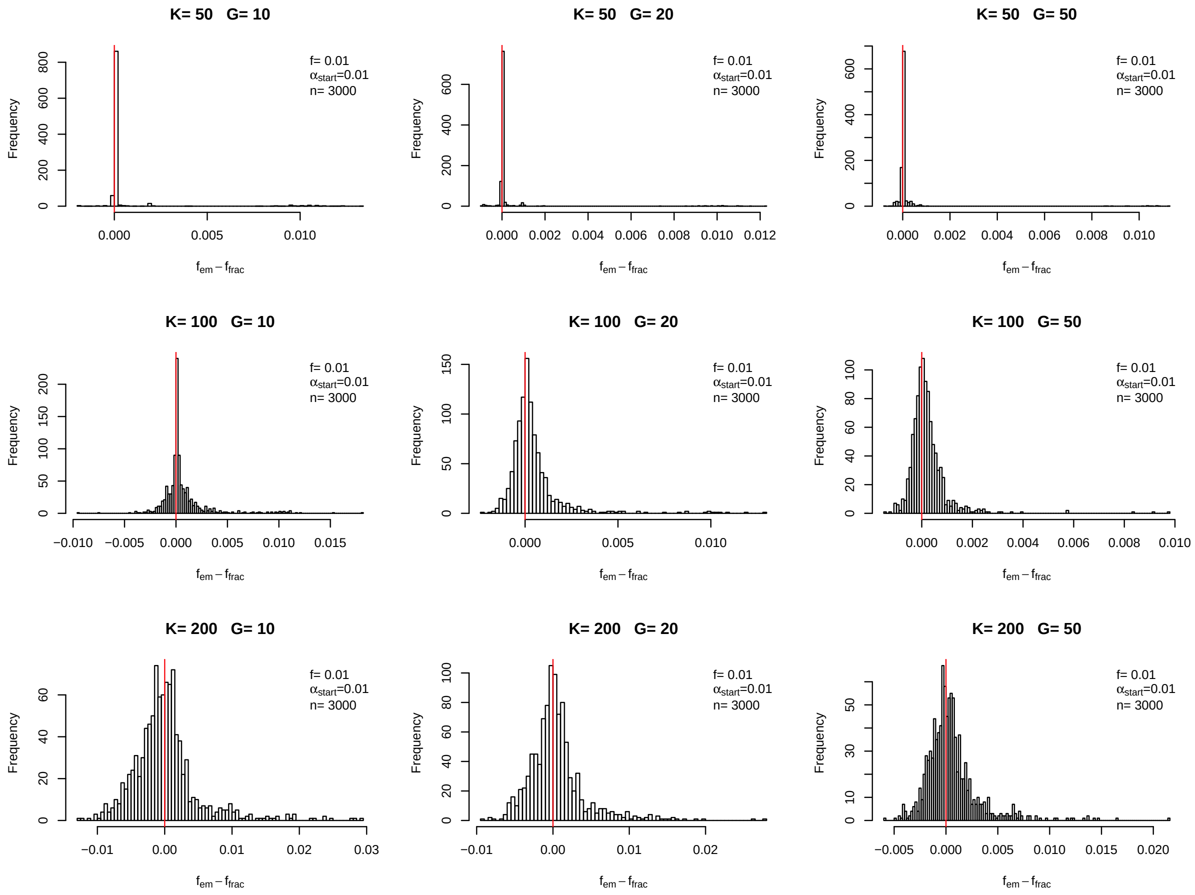

Figure S7: The effects of (K, G) on the estimation accuracy of $\hat{f}_{\mathrm{em}}$ as an estimator of f_{frac}, $(n, f, \alpha_{\mathrm{start}}) = (3000, 0.01, 0.01)$.

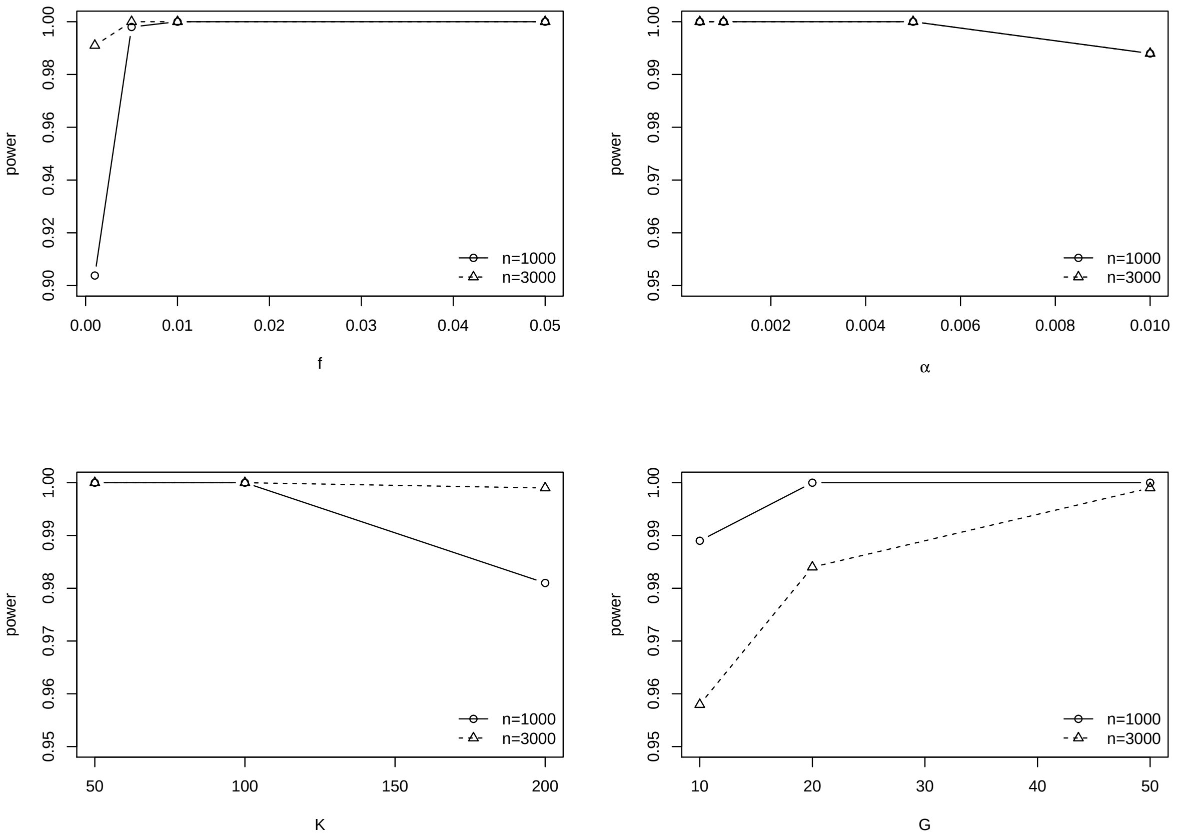

Figure S8: The power of SNP detection at a type I error of 0.05 using cases and controls together, $(K, G, f, \alpha, \lambda) = (100, 20, 1, 0.5\%, 1.2)$.

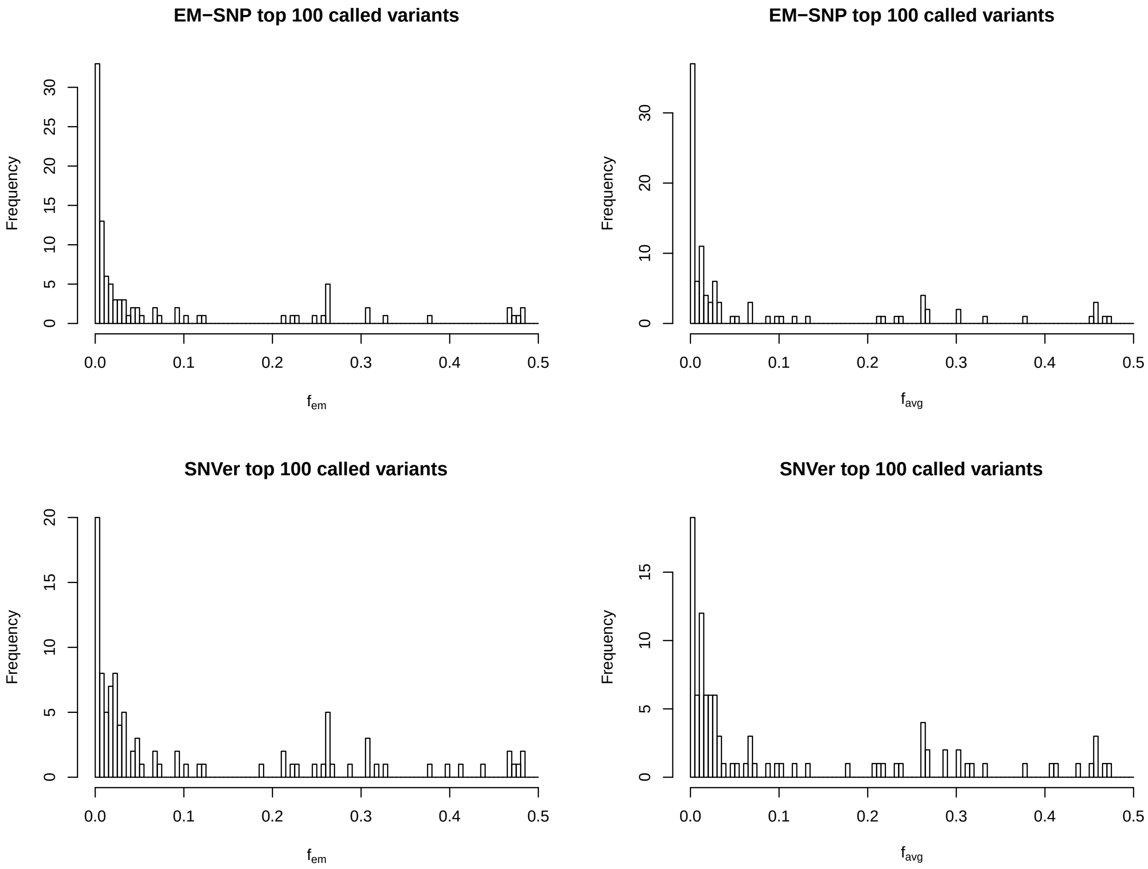

Figure S9: Allele frequency spectrum of the top 100 variants called by EM-SNP and SNVer.

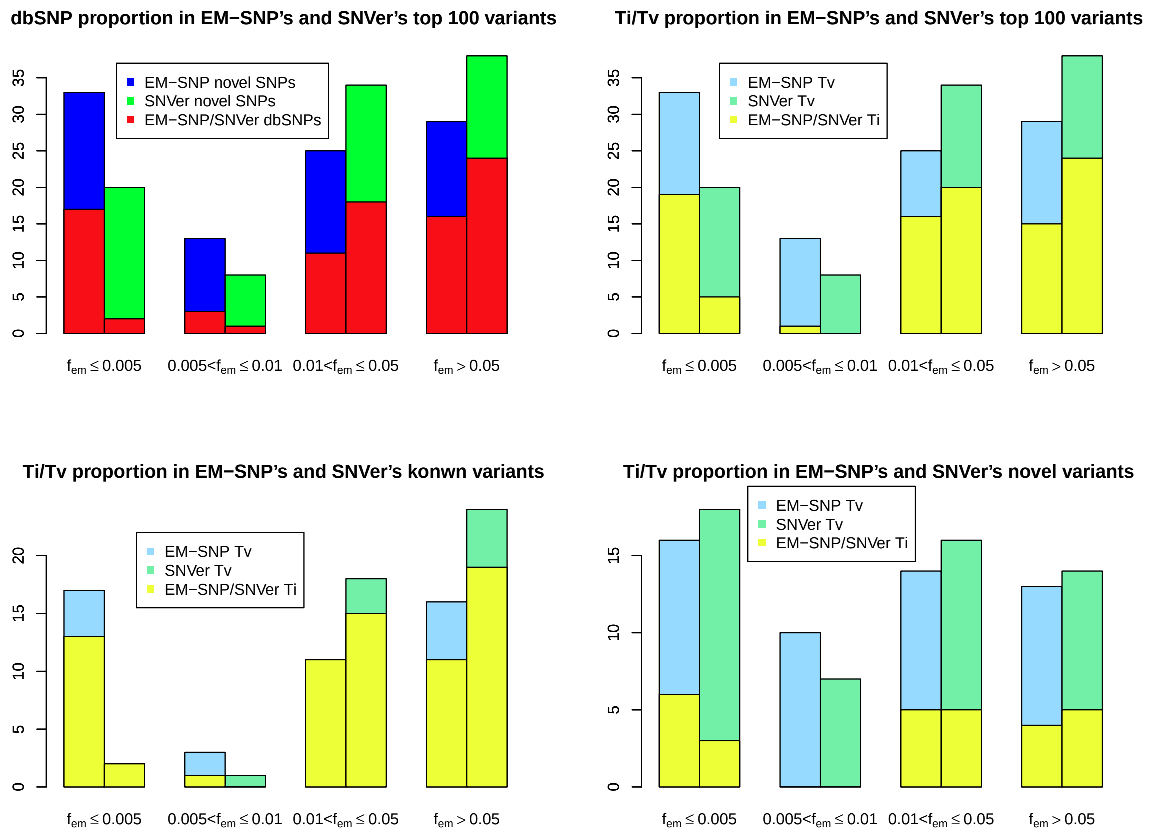

Figure S10: dbSNP proportion and Ti/Tv proportion of the top 100 variants called by EM-SNP and SNVer, grouped by allele frequency level.

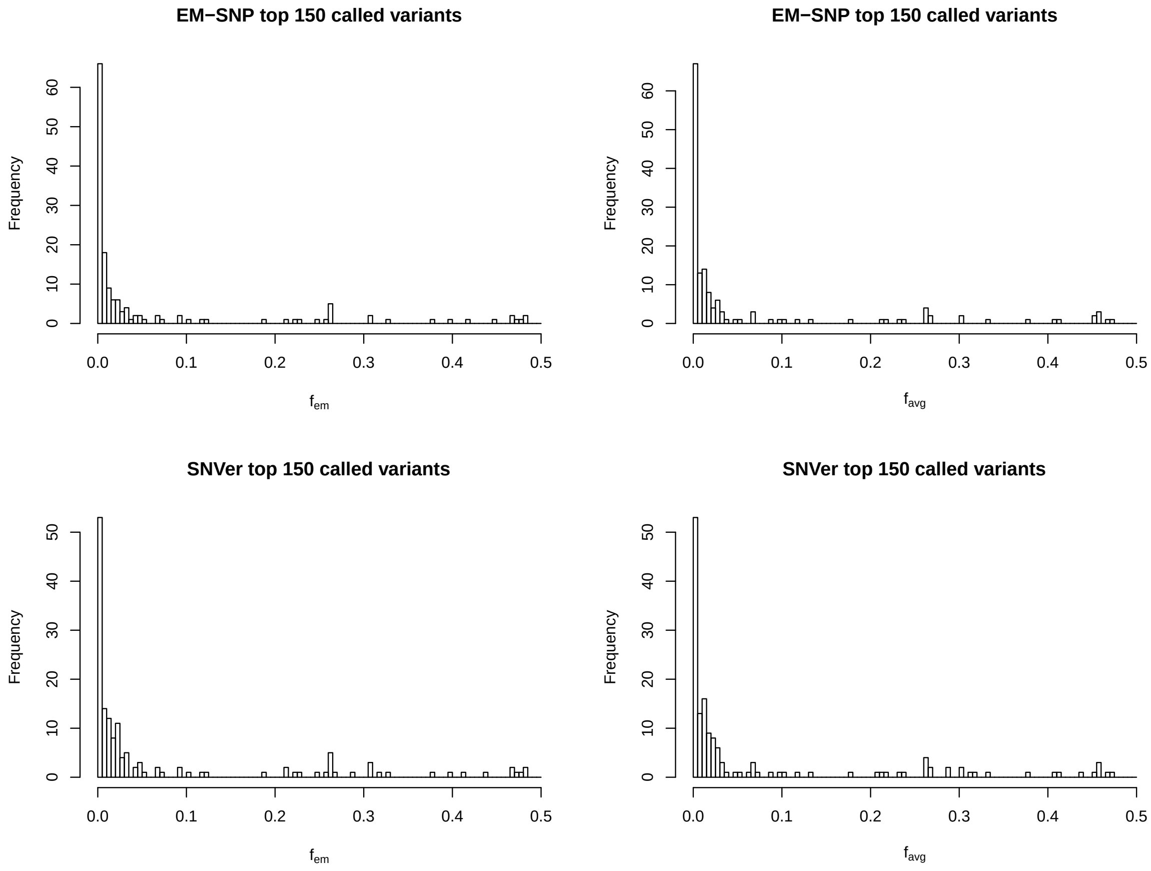

Figure S11: Allele frequency spectrum of the top 150 variants called by EM-SNP and SNVer.

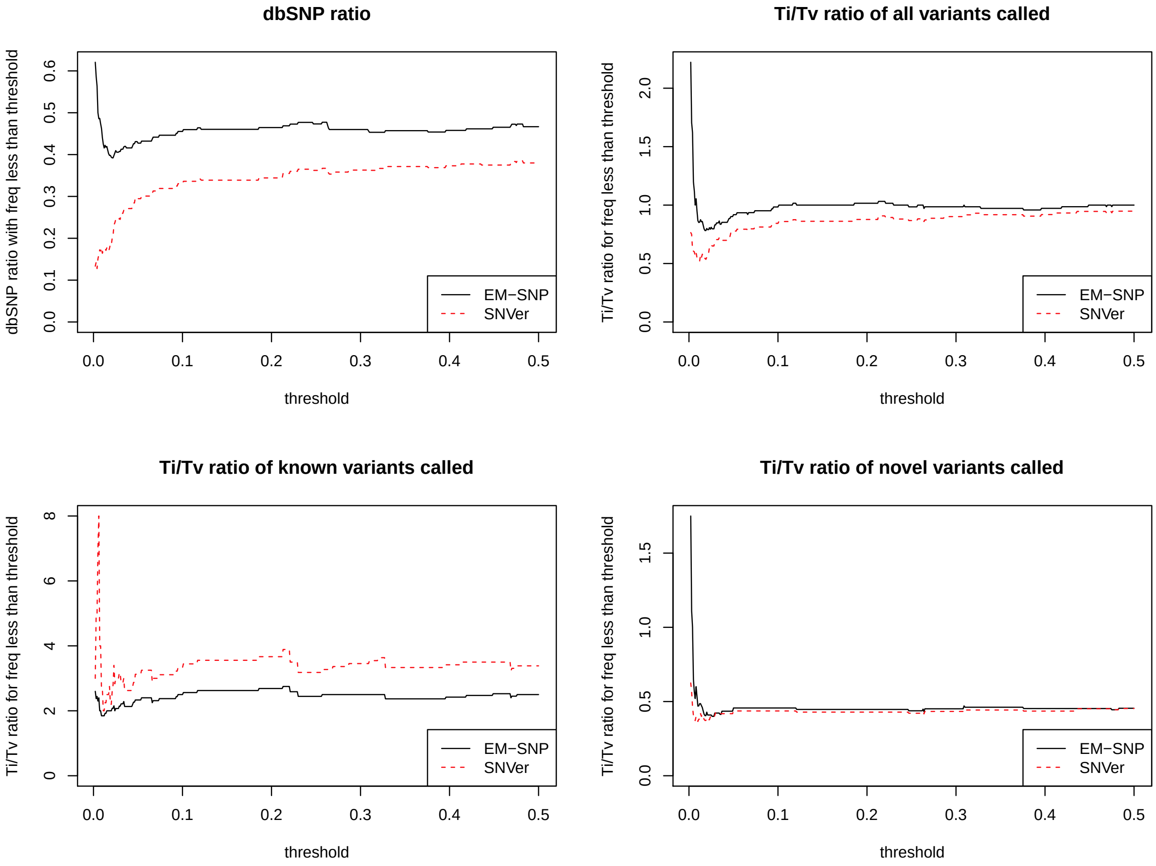

Figure S12: The dbSNP ratio and Ti/Tv ratio of the top 150 variants called by EM-SNP and SNVer.

Supplementary Tables

	$\alpha = 0.05\%$		$\alpha = 0.1\%$		$\alpha = 0.5\%$		$\alpha = 1\%$	
f	MSE	Cg	MSE	Cg	MSE	Cg	MSE	Cg
0.1%	6.3e-07	3.7e-08	6.4e-07	1.2e-07	1.5e-06	9.3e-07	3.6e-06	3.2e-06
0.5%	3.4e-06	1.1e-07	3.4e-06	1.6e-07	4.1e-06	9.6e-07	5.1e-06	1.5e-06
1%	7.4e-06	3.8e-07	7.5e-06	5.1e-07	8.1e-06	1.4e-06	9.1e-06	2.7e-06
5%	3.3e-05	9.4e-06	3.4e-05	9.6e-06	3.8e-05	1.2e-05	4.9e-05	2.0e-05

Table S1: The values of $\sigma_m^2(5)$ (defined in terms of MSE and Cg) for $\hat{f}_{\text{em}}$ as an estimator of f or f_{frac} for different values of f and α, $(K, G, n) = (100, 10, 3000)$.

$\sigma_m^2(10)$	$\alpha = 0.05\%$		$\alpha = 0.1\%$		$\alpha = 0.5\%$		$\alpha = 1\%$	
f	MSE	Cg	MSE	Cg	MSE	Cg	MSE	Cg
0.1%	4.9e-07	3.7e-08	4.8e-07	1.2e-07	7.9e-07	2.2e-07	1.1e-06	4.0e-07
0.5%	2.4e-06	1.1e-07	2.4e-06	1.5e-07	2.9e-06	6.6e-07	3.3e-06	8.6e-07
1%	5.2e-06	3.6e-07	5.2e-06	4.9e-07	5.7e-06	1.4e-06	6.2e-06	2.2e-06
5%	2.4e-05	8.7e-06	2.4e-05	8.8e-06	2.6e-05	1.1e-05	3.2e-05	1.3e-05

Table S2: The values of $\sigma_m^2(10)$ (defined in terms of MSE and Cg) for $\hat{f}_{\text{em}}$ as an estimator of f or f_{frac} for different values of f and α, $(K, G, n) = (100, 10, 3000)$.

	$G = 10$		$G = 20$		$G = 50$	
K	MSE	Cg	MSE	Cg	MSE	Cg
50	3.3e-05	1.1e-05	1.5e-05	4.3e-06	5.5e-06	2.0e-06
100	5.3e-05	4.3e-05	4.5e-05	4.0e-05	1.8e-05	1.6e-05
200	5.2e-05	5.4e-05	5.9e-05	5.9e-05	3.8e-05	3.7e-05

Table S3: The values of MSE and Cg for $\hat{f}_{\text{em}}$ as an estimator of f and f_{frac}, respectively, for different values of (K, G), $(n, f, \alpha_{\text{start}}) = (1000, 0.01, 0.01)$.

K	$G = 10$ MSE	$G = 10$ Cg	$G = 20$ MSE	$G = 20$ Cg	$G = 50$ MSE	$G = 50$ Cg
50	2.4e-05	4.2e-06	1.4e-05	2.6e-06	5.1e-06	1.4e-06
100	1.6e-05	5.6e-06	7.6e-06	2.4e-06	2.8e-06	6.8e-07
200	2.5e-05	2.4e-05	1.6e-05	1.4e-05	7.5e-06	6.8e-06

Table S4: The values of MSE and Cg for $\hat{f}_{\text{em}}$ as an estimator of f and f_{frac}, respectively, for different values of (K, G), $(n, f, \alpha_{\text{start}}) = (3000, 0.01, 0.01)$.

	G = 10			G = 20			G = 50		
K	50	100	200	50	100	200	50	100	200
$\sigma^2_{m,\text{MSE}}(5)$	1.8e-05	2.6e-05	2.8e-05	8.8e-06	1.9e-05	3.3e-05	3.0e-06	6.8e-06	1.6e-05
$\sigma^2_{m,\text{Cg}}(5)$	5.3e-06	2.1e-05	2.8e-05	1.6e-06	1.8e-05	3.4e-05	5.0e-07	5.7e-06	1.6e-05
$\sigma^2_{m,\text{MSE}}(10)$	1.2e-05	1.8e-05	1.8e-05	6.2e-06	1.2e-05	2.1e-05	2.1e-06	4.1e-06	9.2e-06
$\sigma^2_{m,\text{Cg}}(10)$	4.1e-06	1.5e-05	1.9e-05	1.3e-06	1.2e-05	2.2e-05	5.1e-07	3.2e-06	8.9e-06

Table S5: The modified mean-squared-error $\sigma^2_m(5)$ and $\sigma^2_m(10)$ of the estimated values of f for different numbers of chromosomes in a pool K and numbers of pools G, $(n, f, \alpha_{\text{start}}) = (1000, 0.01, 0.01)$.

	G = 10			G = 20			G = 50		
K	50	100	200	50	100	200	50	100	200
$\sigma^2_{m,\text{MSE}}(5)$	1.4e-05	9.1e-06	1.1e-05	7.2e-06	4.1e-06	6.7e-06	2.7e-06	1.5e-06	2.9e-06
$\sigma^2_{m,\text{Cg}}(5)$	2.3e-06	2.7e-06	1.0e-05	1.1e-07	9.2e-07	6.0e-06	1.9e-08	2.9e-07	2.6e-06
$\sigma^2_{m,\text{MSE}}(10)$	1.0e-05	6.2e-06	7.5e-06	5.0e-06	2.8e-06	4.2e-06	1.9e-06	1.1e-06	1.8e-06
$\sigma^2_{m,\text{Cg}}(10)$	1.2e-06	2.2e-06	7.5e-06	1.2e-07	8.1e-07	4.0e-06	1.7e-08	2.6e-07	1.7e-06

Table S6: The modified mean squared error $\sigma^2_m(5)$ and $\sigma^2_m(10)$ of the estimated values of f for different numbers of chromosomes in a pool K and numbers of pools G, $(n, f, \alpha_{\text{start}}) = (1000, 0.01, 0.01)$.

variants called	$f_{\text{EM}} < 0.2\%$			$f_{\text{avg}} < 0.2\%$		
	# total	# dbSNP	# novel	# total	# dbSNP	# novel
		a) Top 100 SNPs				
EM-SNP	11	9	2	13	11	2
SNVer	10	1	9	10	0	10
		b) Top 150 SNPs				
EM-SNP	29	18	11	30	22	8
SNVer	30	4	26	26	2	24

Table S7: The total number of SNPs (2nd and 5th columns), the number of SNPs in the dbSNP database (3rd and 6th columns), and the number of novel SNPs (4th and 7th columns) among the top a) 100 and b) 150 EM-SNP or SNVer called SNPs with minor allele frequency at most 0.2% according to either f_{em} or f_{avg}. The dbSNP ratio for the SNPs called by EM-SNP is much higher than that for SNVer.

variants called	total		known(dbSNP)		novel	
	#Ti	#Tv	#Ti	#Tv	#Ti	#Tv
b) Top 100 SNPs						
EM-SNP	9	2	7	2	2	0
SNVer	3	7	1	0	2	7
b) Top 150 SNPs						
EM-SNP	20	9	13	5	7	4
SNVer	13	17	3	1	10	6

Table S8: The number of transitions (Ti) (2nd, 4th and 6th columns) and the number of transversions (Tv) (3rd, 5th and 7th columns) among the top a) 100 and b) 150 EM-SNP or SNVer called SNPs with minor allele frequency at most 0.2% according to either f_{em} or f_{avg}. The Ti/Tv ratio for the SNPs called by EM-SNP is much higher than that for SNVer.
